# Energy-Dependent Response and Multiscale Damage of Hybrid Unidirectional-Tape/Woven-Fabric Composite Laminates Under Low-Velocity Impact: Experimental Characterization and Numerical Analysis

**DOI:** 10.3390/polym18192414

**Published:** 2026-10-02

**Authors:** Yu Feng, Rui Shen, Qingqiang Zeng, Zhuogang Fu, Binlin Ma

**Affiliations:** 1 School of Aviation Engineering, Air Force Engineering University, Xi’an 710038, China; fengyu1609@163.com (Y.F.);; 2 School of Ordnance Science and Technology, Xi’an Technological University, Xi’an 710021, China

**Keywords:** low-velocity impact, hybrid UD/WF composite laminate, multiscale damage, progressive damage model, finite element analysis

## Abstract

Despite growing interest in hybrid unidirectional-tape/woven-fabric (UD/WF) composite laminates, the relationship between impact energy, macroscopic response, and multiscale damage evolution under low-velocity impact remains insufficiently understood. To address this issue, hybrid UD/WF composite laminates were investigated under low-velocity impacts of 10, 17, and 25 J through experiments and numerical simulations. Phased-array ultrasonic testing, micro-computed tomography (μCT), and cross-sectional microscopy were used to characterize multiscale damage, while a continuum-damage-mechanics-based progressive damage model was developed to capture intralaminar failure in the UD and woven-fabric plies and interlaminar delamination. The results showed a clear energy dependence of the impact response. The Hertzian failure load varied only slightly with impact energy, whereas the peak load, maximum displacement, and dissipated energy increased from 5.47 kN, 3.77 mm, and 1.91 J at 10 J to 10.08 kN, 5.51 mm, and 5.33 J at 25 J, respectively. Increasing impact energy intensified surface indentation and matrix cracking along the warp and weft directions. The projected delamination area increased from 484.15 mm^2^ at 10 J to 923.78 mm^2^ at 17 J and 987.70 mm^2^ at 25 J, indicating a reduced rate of in-plane delamination growth at higher energies. Notably, from 17 to 25 J, the dissipated energy nearly doubled, whereas the projected delamination area increased by only 6.9%, indicating that the increase in energy dissipation was not accompanied by a proportional increase in the in-plane extent of delamination. At 25 J, internal damage was concentrated near the impact region and was dominated by delamination and matrix cracking, with no extensive fiber fracture in the UD plies. The numerical model reproduced the global impact response, indentation depth, and delamination extent with reasonable accuracy and further revealed nonuniform attenuation of delamination through the laminate thickness. These findings clarify the non-proportional evolution of energy dissipation and in-plane delamination growth with increasing impact energy and provide a basis for impact damage assessment and impact-resistant design of hybrid UD/WF laminates.

## 1. Introduction

Carbon fiber-reinforced polymer (CFRP) composites have been widely used in aerospace, marine, and automotive structures because of their high specific strength and stiffness, excellent fatigue resistance, and corrosion resistance [1,2,3,4,5,6]. Depending on the reinforcement architecture and stacking configuration, laminated composites can be broadly classified into unidirectional (UD), two-dimensional woven, three-dimensional woven, and hybrid composite laminates [7]. Among them, hybrid unidirectional-tape/woven-fabric (UD/WF) laminates combine the high in-plane load-bearing capability of UD plies with the improved surface damage resistance and damage tolerance provided by woven-fabric plies, making them attractive for aerospace structural applications [8,9]. However, composite structures are inevitably exposed to low-velocity impacts caused by events such as tool drops and runway debris during manufacturing, service, and maintenance [10]. Such impacts may leave only minor surface indentations while inducing internal matrix cracking, interlaminar delamination, and even fiber damage, which can significantly reduce the residual load-bearing capacity and compromise structural safety. Therefore, a comprehensive understanding of the low-velocity impact response and internal damage characteristics of hybrid UD/WF laminates is essential for reliable damage assessment and impact-resistant design.

Considerable efforts have been devoted to characterizing and predicting the low-velocity impact behavior of composite laminates. Nondestructive evaluation techniques, including ultrasonic testing [11,12,13], infrared thermography [14], and X-ray computed tomography (CT) [15], together with destructive methods such as thermal deply [16], have been widely employed to characterize impact-induced damage. These studies have shown that low-velocity impact damage generally involves multiple interacting failure modes, among which matrix cracking and interlaminar delamination are predominant, with fiber damage becoming increasingly significant as the impact severity increases [17,18]. In parallel, progressive damage models based on continuum damage mechanics (CDM) have been extensively used to predict impact response by describing the initiation and evolution of different intralaminar failure modes [19,20,21]. When combined with cohesive zone models (CZMs), such approaches can further capture the initiation and propagation of interlaminar delamination. The integration of multiscale damage characterization with progressive damage modeling therefore provides an effective framework for linking global impact response to internal damage mechanisms.

In recent years, increasing attention has been directed toward the impact behavior of hybrid UD/WF laminates. Ma et al. [8] compared the low-velocity impact responses of UD, woven-fabric, and hybrid UD/WF laminates and demonstrated that the stacking architecture strongly affects both impact response and damage characteristics. Wang et al. [9] further investigated the compression–compression fatigue behavior of hybrid UD/WF laminates after low-velocity impact and established relationships among impact indentation, delamination damage, and fatigue degradation. Liu et al. [22,23,24] conducted experimental and numerical studies on the impact, compression-after-impact, and crushing behavior of hybrid UD/WF laminates and developed corresponding damage prediction models based on continuum damage mechanics and fracture mechanics. Mahmoud et al. [25] investigated the impact damage mechanisms of hybrid UD/WF composites through combined experimental and numerical approaches, further demonstrating the important role of hybrid layup configurations in governing impact damage. In addition, Rezasefat et al. [26] showed that hybridization strategy, impact side, and stacking sequence can significantly influence impact stiffness and its degradation in interply hybrid laminates. These studies confirm that hybrid design can substantially alter the impact response and damage mechanisms of composite laminates and provide an important basis for their numerical prediction.

Although previous studies have investigated the low-velocity impact behavior of hybrid UD/WF composite laminates, the quantitative relationship between energy dissipation and damage morphology, as well as the through-thickness evolution of delamination, remains insufficiently understood. In this study, the impact damage behavior of hybrid UD/WF laminates is systematically investigated by combining drop-weight impact testing, phased-array ultrasonic C-scan, X-ray μCT characterization, and progressive damage simulation. The main contributions of this work are summarized as follows. First, the energy-dependent evolution of impact response and damage characteristics is quantitatively analyzed, revealing that the increase in dissipated energy does not necessarily correspond to a proportional increase in projected delamination area. Second, the interface-by-interface distribution of delamination is numerically resolved, revealing the nonuniform attenuation behavior of delamination through the laminate thickness. Finally, an experimentally validated progressive damage model is established to correlate the macroscopic impact response with the underlying damage mechanisms in hybrid UD/WF laminates.

## 2. Materials and Experimental Methods

### 2.1. Materials and Laminate Configuration

**Figure 1:**
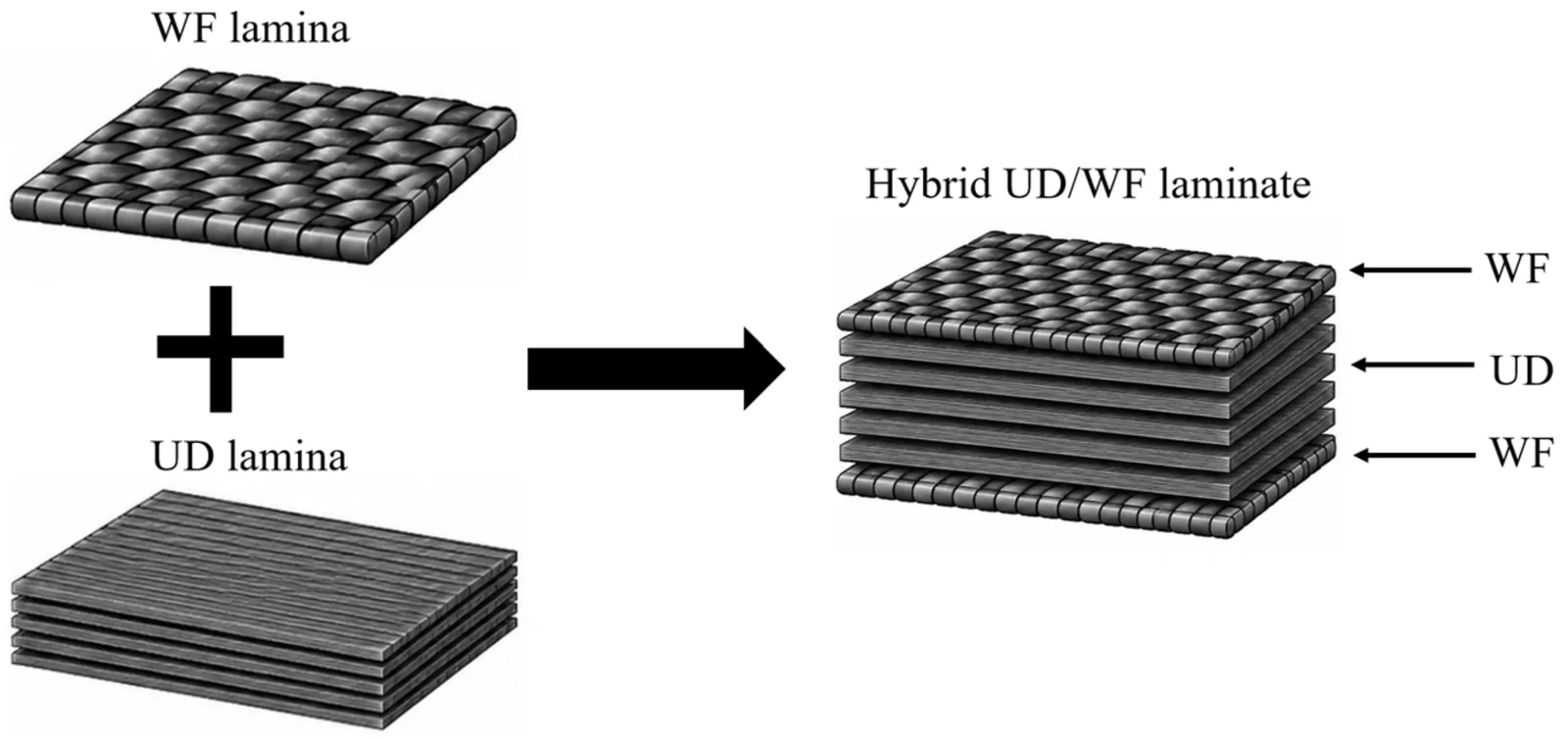
Schematic of the hybrid UD/WF laminate specimen and stacking configuration.

The investigated specimens were hybrid unidirectional-tape/woven-fabric (UD/WF) composite laminates, as illustrated in Figure 1. The laminates were manufactured by Aviation Industry Corporation of China (AVIC, Beijing, China) using Toray T700SC carbon fibers and a CB646 toughened epoxy resin system. Two types of prepreg, namely UD prepreg and 12K plain-weave fabric prepreg, were employed. The UD prepreg had an areal density of 238 g/m^2^ and a resin content of 37%, with a cured ply thickness of 0.142 mm. The WF prepreg had an areal density of 690 g/m^2^ and a resin content of 42%, with a cured ply thickness of 0.422 mm. The main mechanical properties of the UD and WF plies are listed in Table 1 and Table 2, respectively, and were measured and provided by the manufacturer.

The laminate stacking sequence was [45*/0/0/45/90/-45/0/45/90/-45/0]_s_, where the superscript “*” denotes a woven-fabric ply and all other plies are UD plies. In this configuration, the UD plies serve as the primary load-bearing layers, while the WF plies are placed on the laminate surfaces. The cured laminates had an average thickness of 3.68 mm, a fiber volume fraction of 61%, and an average density of 1634 kg/m^3^. In accordance with ASTM D7136/D7136M [27], the laminates were cut into rectangular specimens measuring 150 mm × 100 mm to ensure consistent specimen dimensions and boundary conditions for all impact tests.

In Table 1 and Table 2, *E_i_* (*i* = 1, 2, 3) denotes the elastic modulus in the principal material directions; *G_ij_* and *v_ij_* (*i*, *j* = 1, 2, 3) denote the shear modulus and Poisson’s ratio, respectively. *S_ij_* denotes the corresponding shear strength, and *ρ* is the material density. *X_t_*, *Y_t_*, and *Z_t_* represent the tensile strength along the 1-, 2-, and 3-directions, whereas *X_c_* and *Y_c_* represent the compressive strengths in the 1- and 2-directions, respectively. For the UD plies, the 1-, 2-, and 3-directions correspond to the fiber, in-plane transverse, and through-thickness directions, respectively; for the WF plies, the 1- and 2-directions correspond to the warp and weft directions.

### 2.2. Low-Velocity Impact Tests

**Figure 2:**
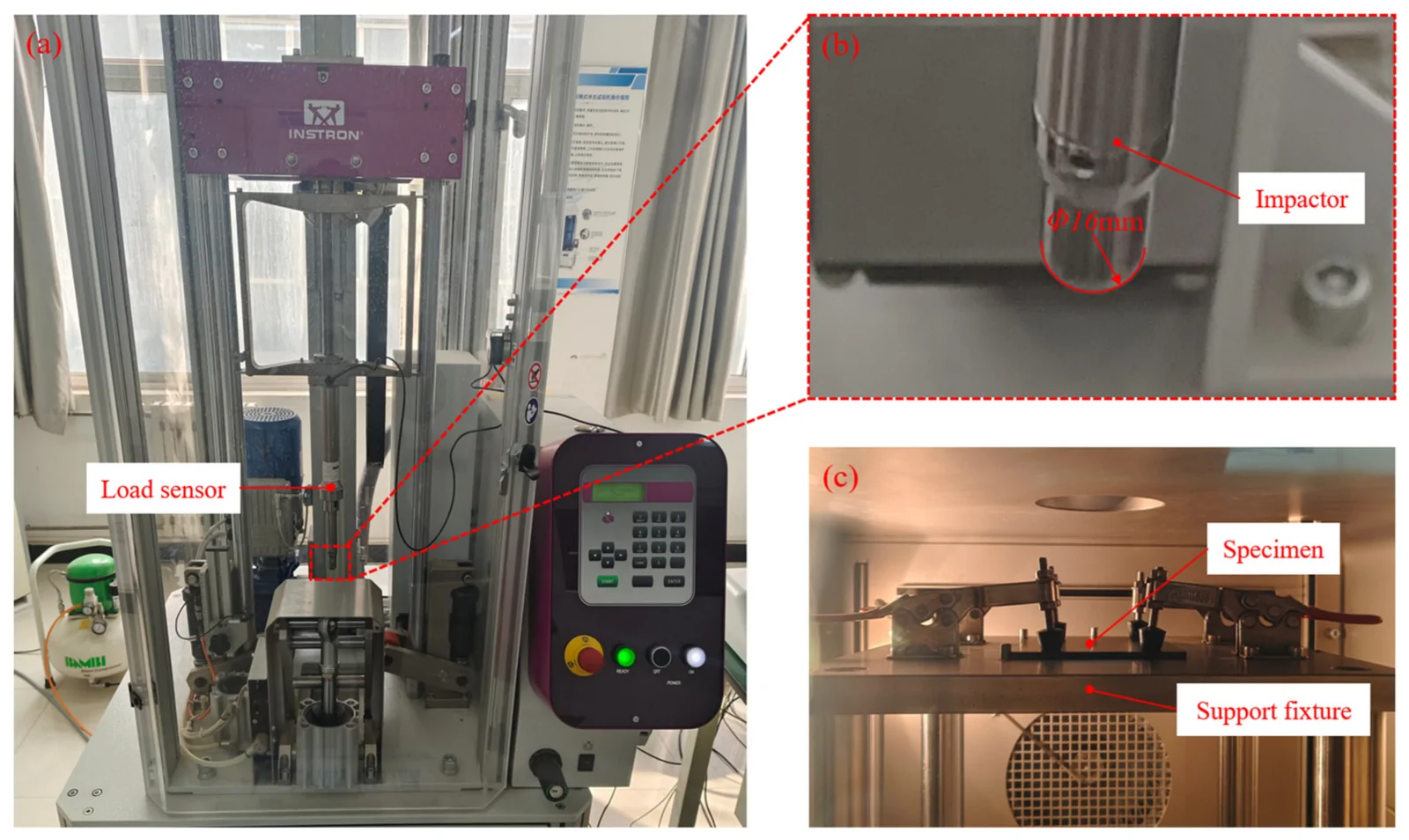
Low-velocity impact test setup: (**a**) drop-weight impact tester; (**b**) hemispherical impactor; and (**c**) specimen fixture.

Low-velocity impact tests were conducted using an Instron Dynatup 9250HV drop-weight impact tester (Instron, Norwood, MA, USA) in accordance with ASTM D7136/D7136M [27], as shown in Figure 2. A hemispherical steel impactor with a diameter of 16 mm and a total mass of 5.607 kg was used. Impact energies of 10, 17, and 25 J were achieved by adjusting the release height of the impactor, with all impacts applied at the center of the specimens. The impact tester was equipped with a pneumatic anti-rebound device to prevent secondary impact. Five specimens were tested at each impact energy.

The specimens were supported by a fixture with a rectangular recess of 150 mm × 100 mm × 4 mm and a central opening of 124 mm × 74 mm (Figure 2c). Four rubber-tipped clamps were used to restrain specimen movement during impact. The load, displacement, and energy responses were recorded throughout each test to characterize the impact behavior at different energy levels.

### 2.3. Multiscale Damage Characterization

**Figure 3:**
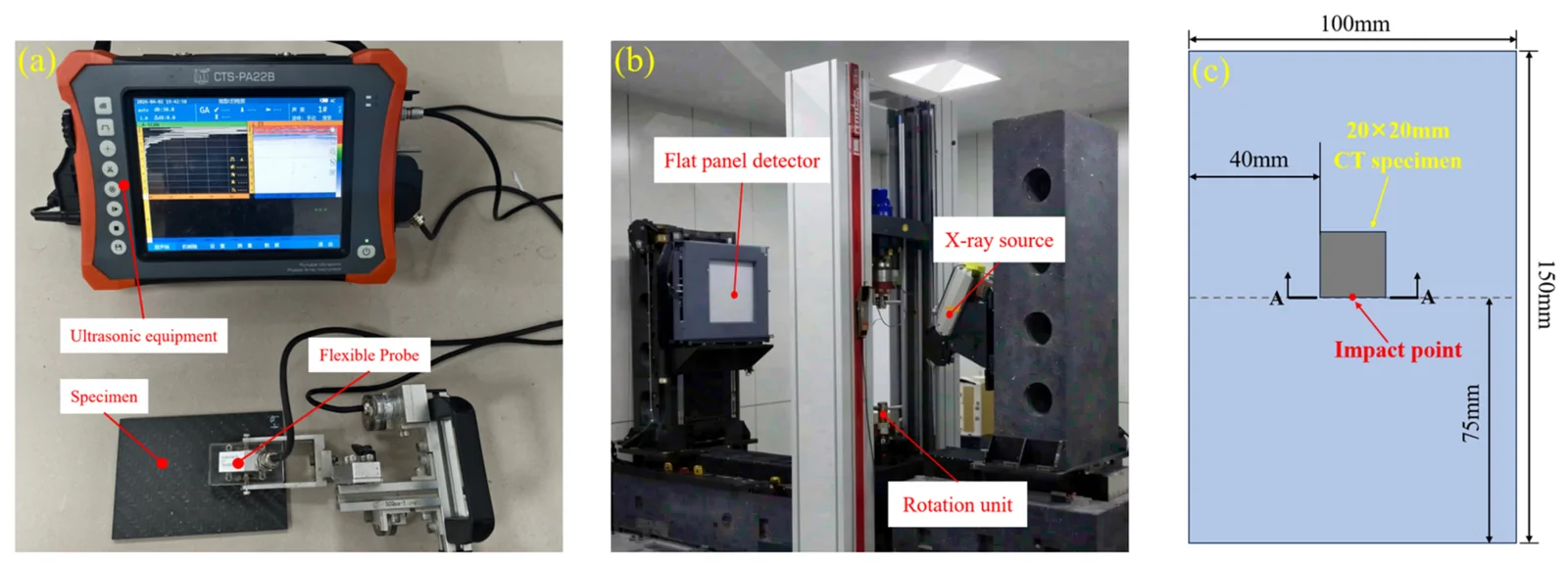
Multiscale damage characterization: (**a**) phased-array ultrasonic testing system; (**b**) micro-CT system; and (**c**) μCT sampling region and cross-sectional observation location.

Post-impact damage was characterized at multiple scales using visual inspection, phased-array ultrasonic testing, X-ray micro-computed tomography (μCT), and cross-sectional optical microscopy observations. First, the impact and back surfaces of each specimen were visually inspected to identify macroscopic damage. Phased-array ultrasonic testing was then performed using a CTS-PA22B system, as shown in Figure 3a, to obtain C-scan images for evaluating the projected extent and distribution of internal damage. During C-scan post-processing, an amplitude gate threshold of 50% of the full-screen height (FSH) was adopted to identify damage indications. Regions with gated echo amplitudes exceeding this threshold were used to determine the projected delamination damage area (DDPA), and the same threshold was applied to all specimens.

The internal damage within the impact region was further examined using a GE micro-CT system [Figure 3b]. Owing to the specimen-size limitation of the μCT system, a 20 mm × 20 mm section centered on the impact location was extracted by waterjet cutting for μCT scanning, as illustrated in Figure 3c.To assess possible damage introduced by waterjet cutting, μCT sections at different locations relative to the cut boundaries and impact region were additionally examined. No discernible damage originating from the waterjet-cut edges was observed within the resolution of the μCT scan. In addition, the damage observed toward the central impact region was cross-checked against the phased-array ultrasonic results obtained before cutting. After scanning, the same specimen was ground and polished along the A–A cross-section indicated in Figure 3c, the polished cross-sections were examined using an optical microscope (DSX1000, Olympus, Tokyo, Japan) at magnifications of ×20–×1000 to characterize microscale damage features, including matrix cracking, interlaminar delamination, and fiber damage.

## 3. Low-Velocity Impact Response and Damage Characteristics

### 3.1. Typical Impact Response and Stage Characteristics

**Figure 4:**
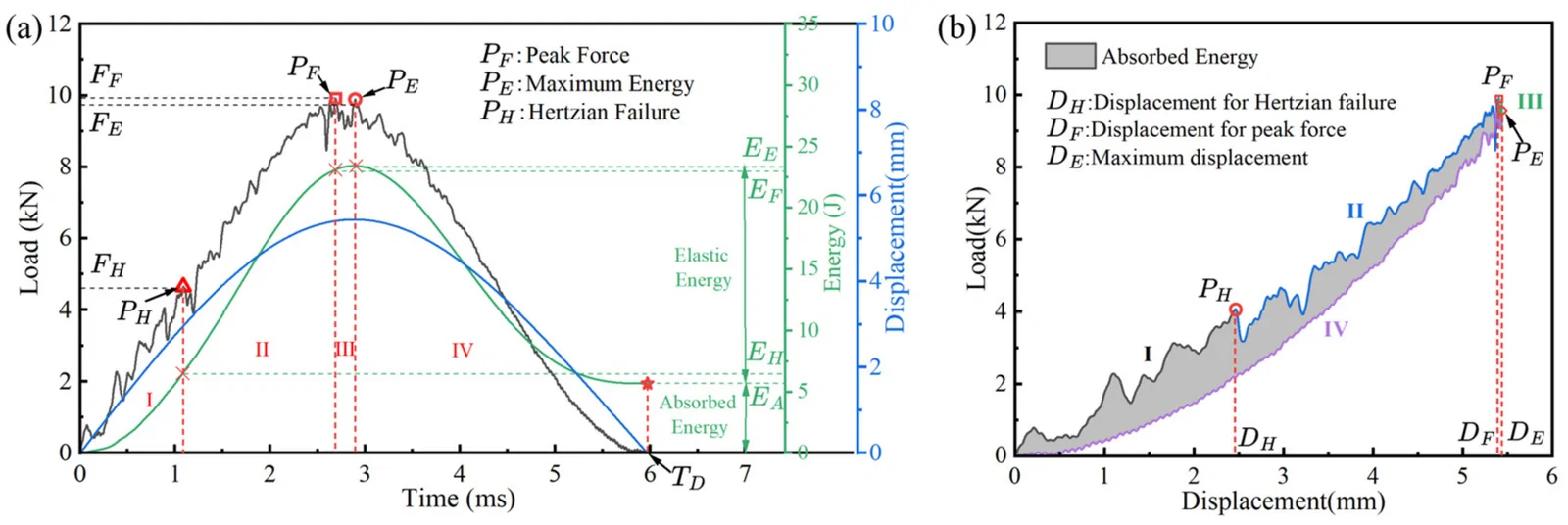
Typical impact response of the hybrid UD/WF laminate under a 25 J impact: (**a**) time histories of load, displacement, and energy; and (**b**) load–displacement response.

Composite laminates generally exhibit a distinct stage-dependent response under low-velocity impact [8]. To illustrate the characteristic impact behavior, the 25 J case, which exhibited relatively pronounced deformation and damage, was selected for detailed analysis, as shown in Figure 4. Based on the variations in the load, displacement, and energy responses, the impact process was divided into four stages. The Hertzian failure point (*P_H_*), which is commonly associated with the onset of impact-induced damage in composite laminates [28], peak-load point (*P_F_*), and load point corresponding to the maximum impact energy (*P_E_*) were identified as characteristic points.

Stage I corresponds to the initial loading stage. Following contact between the impactor and the specimen, the load increases rapidly to *P_H_*, while the load–displacement response remains approximately linear. The small fluctuations observed during this stage are mainly associated with stress-wave propagation and structural vibration.

Stage II represents the nonlinear loading stage. After *P_H_*, the load continues to increase with increasingly pronounced fluctuations, while the load–displacement curve gradually deviates from its initial linear trend, indicating progressive stiffness degradation of the laminate. Combined with the subsequent damage observations, this nonlinear response is associated primarily with the initiation and development of matrix cracking and interlaminar delamination. The stage terminates when the load reaches the peak value at *P_F_*.

Stage III corresponds to the post-peak deformation stage. After *P_F_*, the load generally decreases with continued fluctuations, whereas the specimen displacement continues to increase until *P_E_*, where the impact energy reaches its maximum. This behavior indicates that deformation and energy dissipation continue even after the peak load has been reached. Subsequent damage characterization shows that the laminate remains structurally intact after the 25 J impact, with internal damage dominated by matrix damage and interlaminar delamination and no extensive fiber fracture observed in the UD plies.

Stage IV represents the unloading and rebound stage. Beginning at *P_E_*, the impact energy reaches its maximum and the specimen displacement approaches its maximum value. The impactor subsequently rebounds owing to the elastic recovery of the laminate, and the contact load gradually decreases to zero. The resulting hysteresis loop in the load–displacement curve reflects the irreversible energy dissipation accumulated during the impact event.

The load–displacement response in Figure 4b is consistent with the above stage division. The response is initially approximately linear, followed by a transition to nonlinear loading. After the peak load is reached, the specimen continues to deform until the maximum displacement is attained, after which unloading and rebound occur.

### 3.2. Mechanical Response at Different Impact Energies

Five valid replicate tests were conducted for the UD/WF laminates at each impact energy of 10, 17, and 25 J. The experimental results exhibited low scatter and good repeatability.

#### 3.2.1. Impact Response Curves

**Figure 5:**
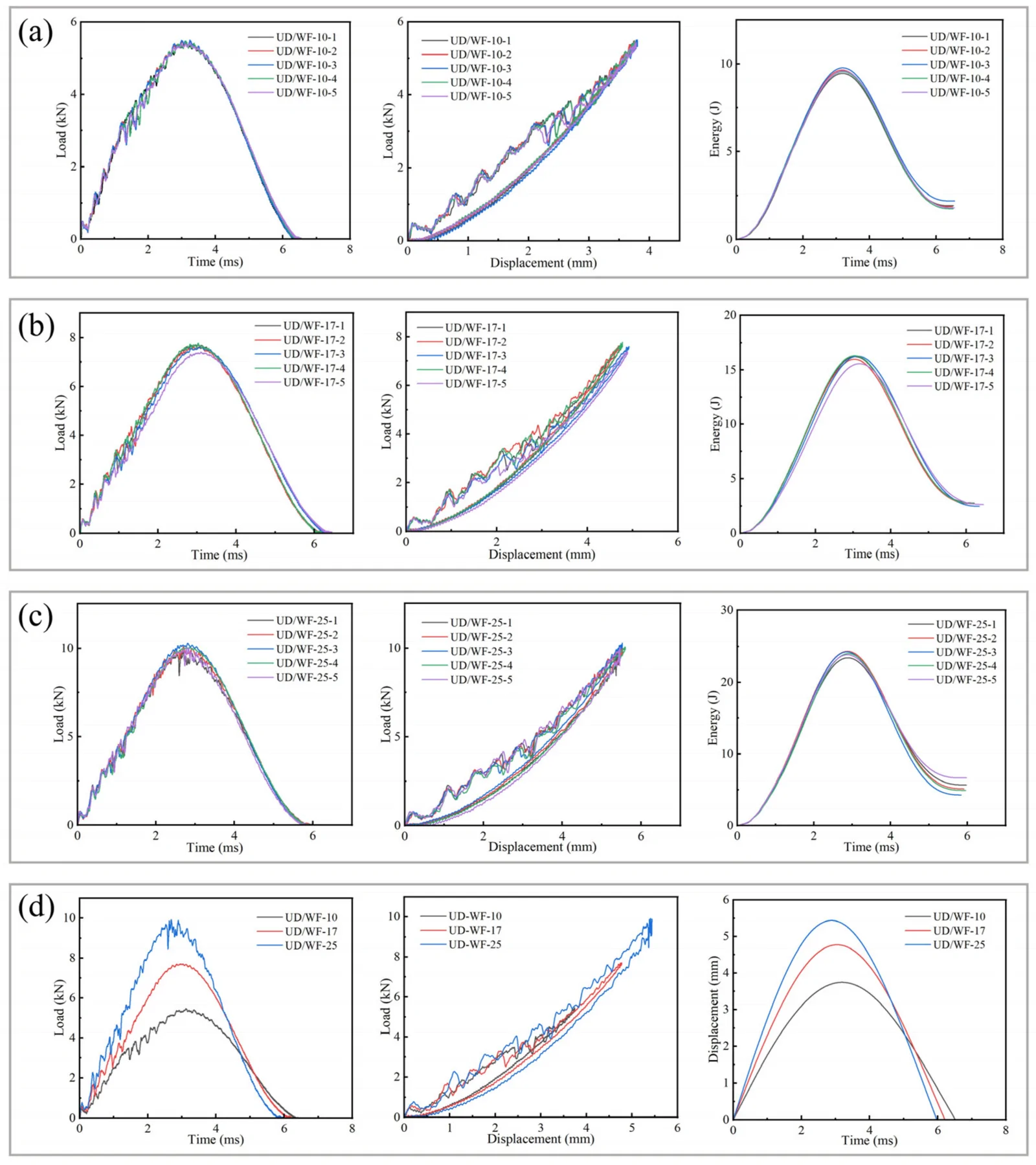
Impact responses of the UD/WF laminates at different impact energies: (**a**) 10 J; (**b**) 17 J; (**c**) 25 J; and (**d**) comparison of the mean response curves.

Figure 5 presents the representative impact responses of the UD/WF laminates at impact energies of 10, 17, and 25 J, together with a comparison of the corresponding mean response curves. At 10 J, the load–time response was relatively smooth, the maximum displacement was limited, and the load–displacement hysteresis loop remained narrow, indicating relatively low deformation and energy dissipation.

As the impact energy increased to 17 and 25 J, both the peak load and maximum displacement increased, accompanied by more pronounced load fluctuations and progressively enlarged load–displacement hysteresis loops. No abrupt load drop was observed at 25 J, indicating that the laminate did not undergo a sudden loss of load-carrying capacity. Therefore, the present experiments establish only a lower bound (>25 J), rather than an exact value, for the complete-failure threshold of the investigated laminate. As the impact energy further increases toward the failure limit, matrix cracking and interlaminar delamination are expected to become more extensive, accompanied by increasing fiber damage. The progressive development of severe fiber fracture and through-thickness damage would eventually lead to a pronounced loss of load-carrying capacity and, at sufficiently high impact energies, penetration or complete laminate failure.

The energy–time curves exhibited a similar energy-dependent trend. The impact energy increased during loading and was partially released during unloading and rebound before reaching a stable residual value, which represents the irreversible energy dissipated during the impact event. The final dissipated energy increased markedly with increasing impact energy, indicating enhanced deformation- and damage-related energy dissipation.

The comparison of the mean response curves in Figure 5d further demonstrates that increasing the impact energy from 10 to 25 J substantially intensified the load and displacement responses, together with increased load fluctuations and larger load–displacement hysteresis loops. These results indicate a pronounced energy dependence of the deformation and energy dissipation behavior of the hybrid UD/WF laminates. The corresponding damage characteristics at different impact energies are discussed in Section 3.3.

#### 3.2.2. Characteristic Response Parameters

**Figure 6:**
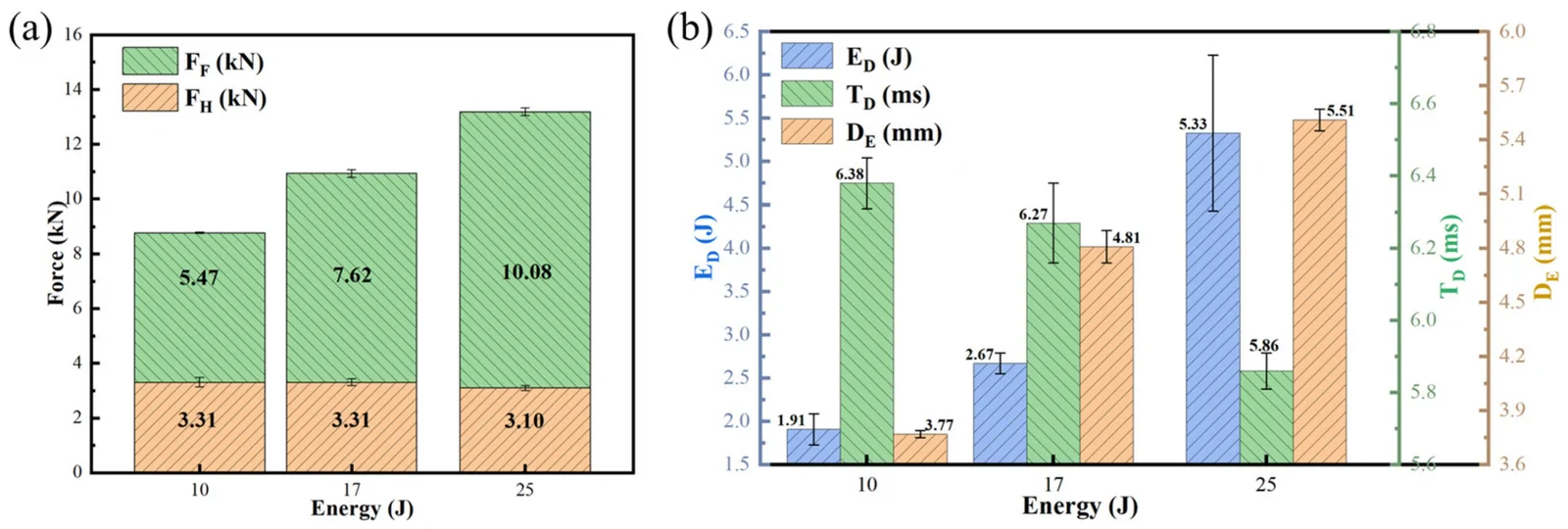
Characteristic response parameters at different impact energies: (**a**) *F_H_* and *F_F_*; (**b**) *E_D_*, *T_D_* and *D_E_*.

Table 3 and Figure 6 summarize the characteristic response parameters of the UD/WF laminates at different impact energies. Within the investigated impact-energy range of 10–25 J, the Hertzian failure load, *F_H_*, was 3.31, 3.31, and 3.10 kN at 10, 17, and 25 J, respectively, showing a relatively limited variation with impact energy. A one-way ANOVA indicated an overall effect of impact energy on *F_H_* (F(2,12) = 4.277, *p* = 0.0396); however, Tukey HSD post hoc comparisons showed no significant pairwise differences at α = 0.05. Although the mean *F_H_* decreased modestly at 25 J, this pairwise difference was not statistically significant.

In contrast, within the investigated impact-energy range of 10–25 J, the peak load *F_F_*, maximum displacement *D_E_*, and dissipated energy *E_D_* increased markedly with increasing impact energy. Specifically, *F_F_* increased from 5.47 kN at 10 J to 10.08 kN at 25 J, while *D_E_* increased from 3.77 to 5.51 mm and *E_D_* from 1.91 to 5.33 J. Meanwhile, the contact duration *T_D_* decreased slightly from 6.38 ms at 10 J to 5.86 ms at 25 J. These results demonstrate that increasing impact energy enhances the load-bearing response, global deformation, and irreversible energy dissipation of the laminate, while slightly reducing the contact duration.

### 3.3. Multiscale Damage Characteristics

#### 3.3.1. Surface Damage Characteristics

**Figure 7:**
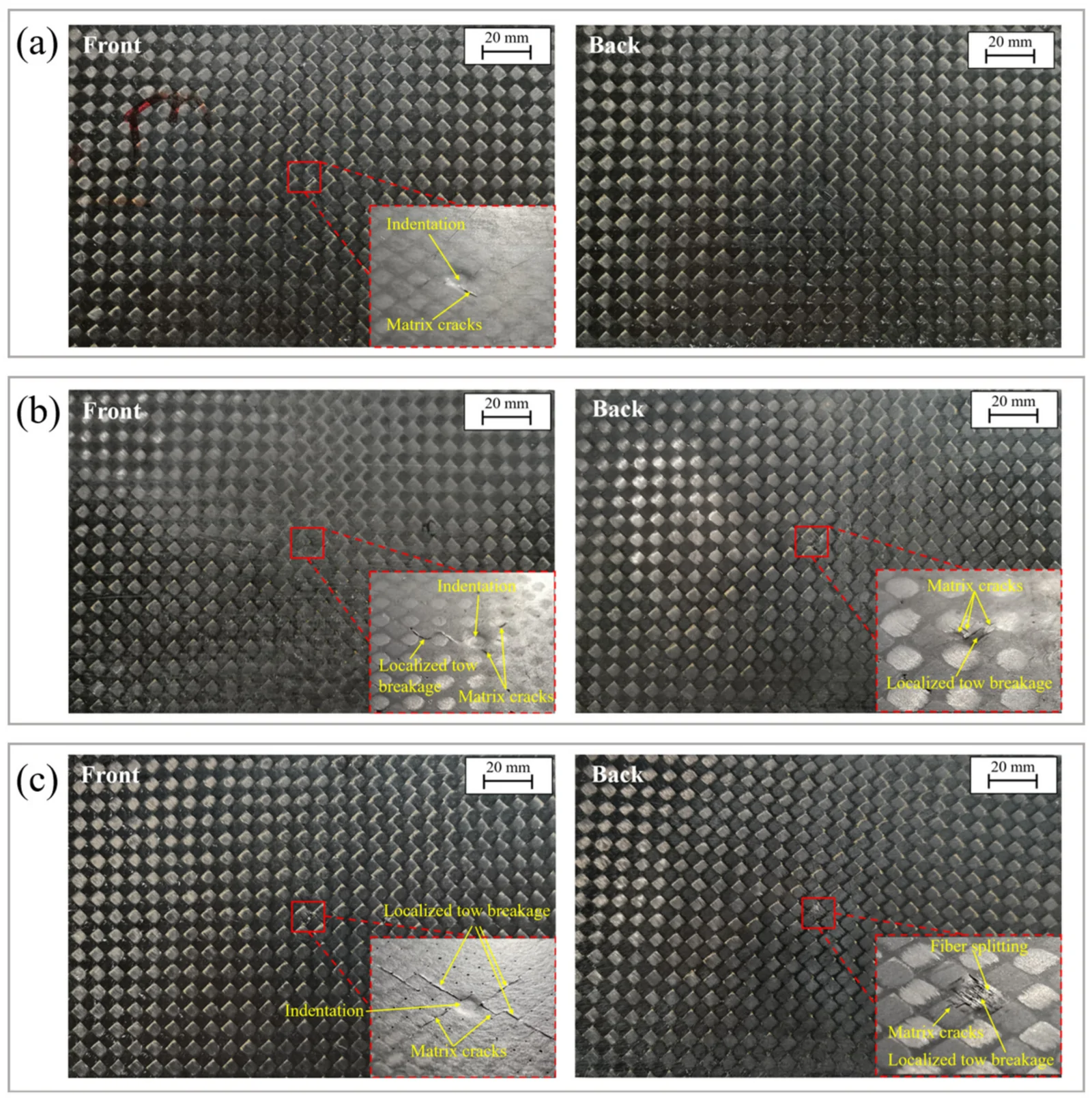
Surface damage morphologies of the UD/WF laminates at different impact energies: (**a**) 10 J; (**b**) 17 J; and (**c**) 25 J.

Figure 7 shows the surface damage morphologies on the impact and back surfaces of the UD/WF laminates at different impact energies. The severity of surface damage increased progressively with increasing impact energy. At 10 J, the impact surface exhibited mainly a localized circular indentation, with no obvious surface matrix cracking, while no visible damage was observed on the back surface. At 17 J, radial cracks began to develop along the warp and weft directions on the impact surface, accompanied by a small number of fine cracks on the back surface and localized tow damage in the surface WF plies. When the impact energy increased to 25 J, both the number and extent of surface cracks increased further, and the back-surface damage became more pronounced, with localized tow breakage observed in the surface WF plies. However, no extensive fiber fracture or through-thickness failure was observed. These observations indicate that the surface WF plies promote a distinct warp- and weft-oriented crack pattern after impact, while maintaining the overall integrity of the laminate under the investigated impact energies.

**Figure 8:**
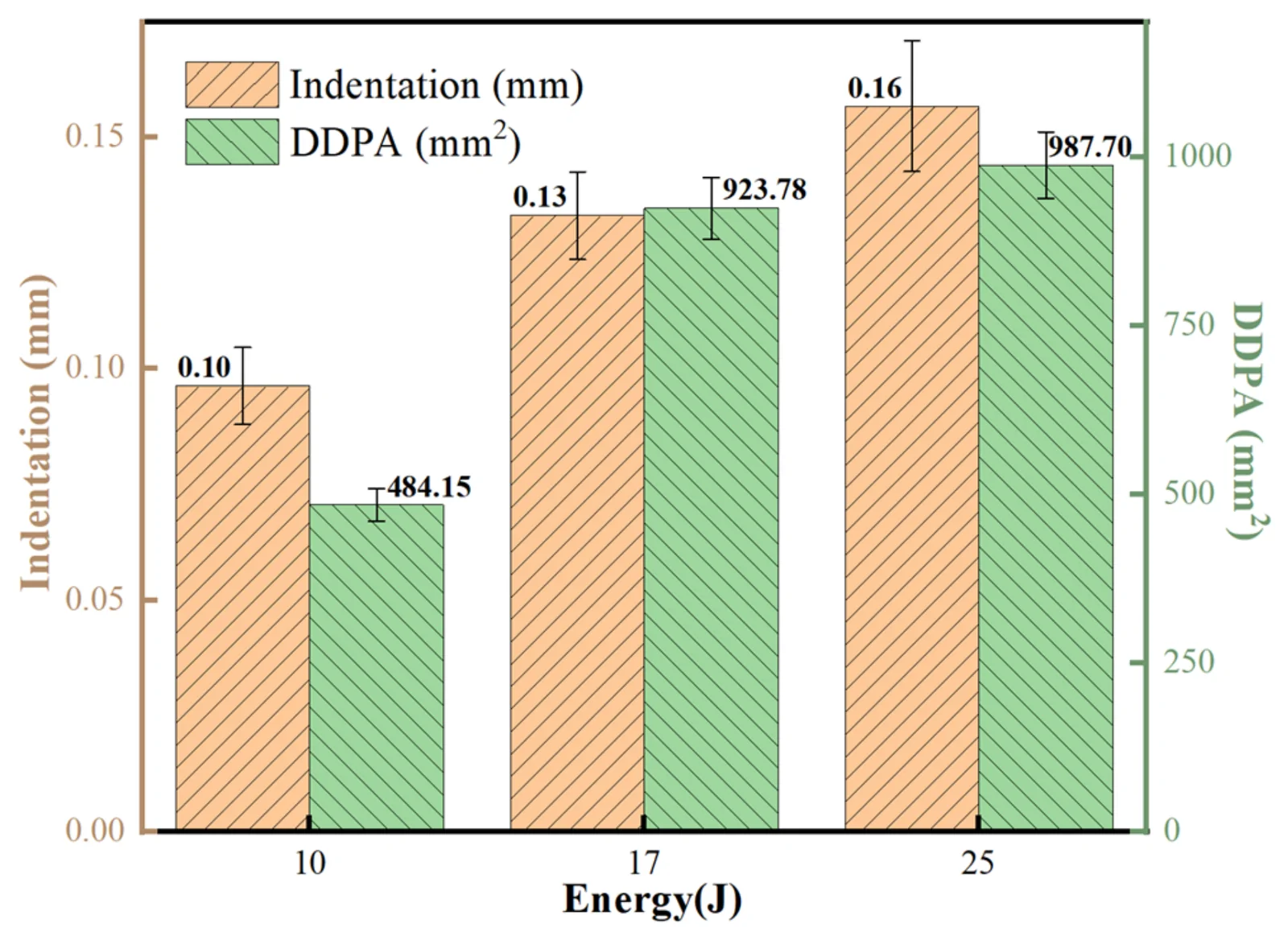
Maximum indentation depth and projected delamination area at different impact energies.

The permanent indentation depth was measured 48 h after impact for each specimen. Although slight dent relaxation may occur during this period, all specimens were measured under the same condition to ensure a consistent comparison among different impact energy levels. The reported indentation depth and DDPA values were obtained from five repeated specimens at each energy level, and the error bars represent the standard deviation.The maximum indentation depths measured 48 h after impact are presented in Figure 8. The mean indentation depths were 0.10, 0.13, and 0.16 mm at 10, 17, and 25 J, respectively. Within the investigated impact-energy range of 10–25 J, the maximum indentation depth increased with impact energy. This trend indicates that increasing impact energy leads to progressively greater irreversible local deformation of the laminate.

#### 3.3.2. Internal Damage Characteristics

**Figure 9:**
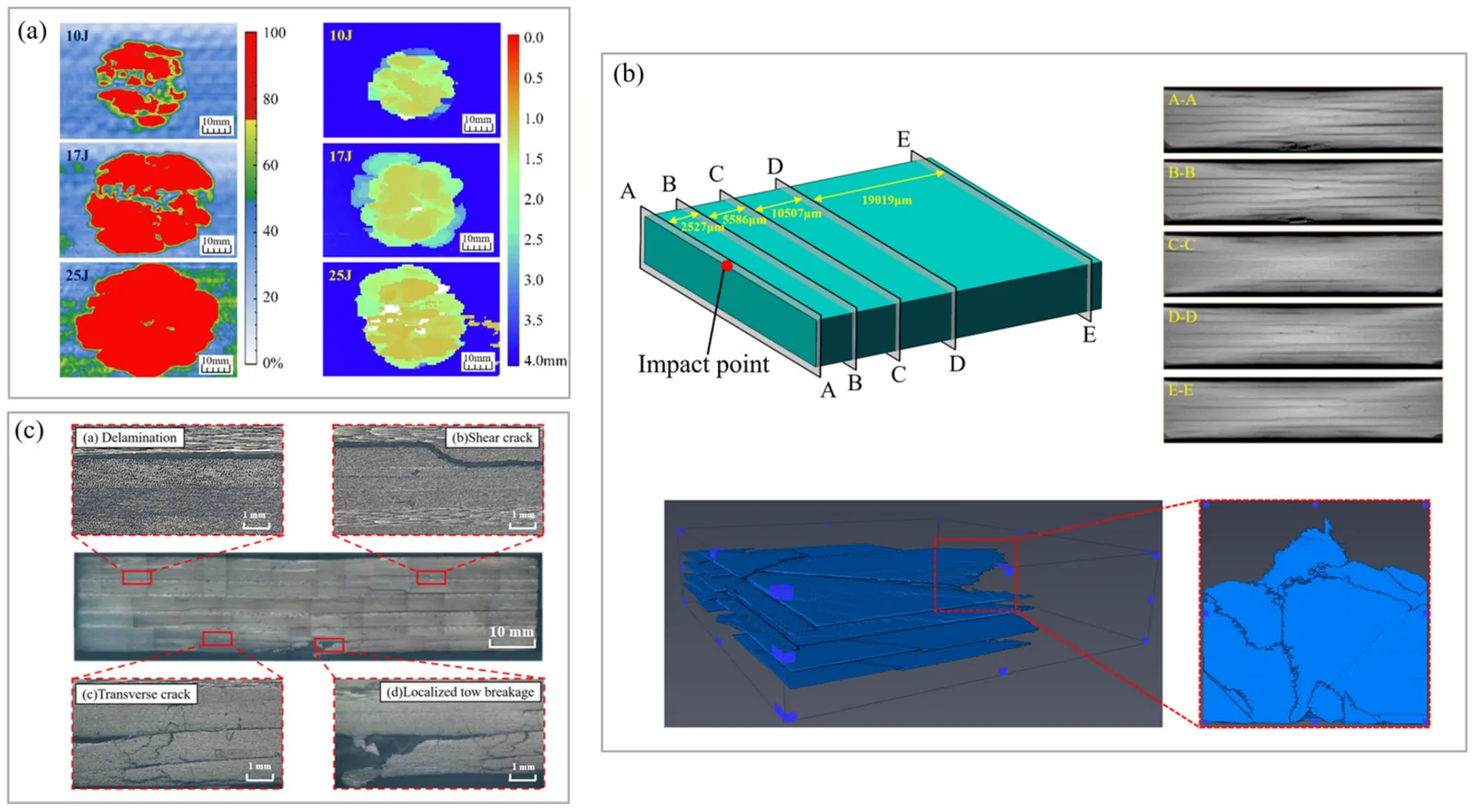
Multiscale characterization of internal damage in the UD/WF laminate: (**a**) phased-array ultrasonic C-scan results; (**b**) X-ray μCT results, where A–E denote different cross-sectional locations; and (**c**) cross-sectional optical microscopy observations.

The phased-array ultrasonic C-scan results are shown in Figure 9a. At all impact energies, the internal damage was concentrated near the impact center and exhibited an approximately circular projected distribution, with the damaged region expanding as the impact energy increased. The corresponding projected delamination damage area (DDPA), shown in Figure 8, was 484.15, 923.78, and 987.70 mm^2^ at 10, 17, and 25 J, respectively. The DDPA increased by approximately 90.8% when the impact energy increased from 10 to 17 J, but by only approximately 6.9% from 17 to 25 J, indicating that the in-plane propagation of delamination tended to slow at higher impact energies.

Notably, the dissipated energy increased from 2.67 J at 17 J to 5.33 J at 25 J, despite the limited increase in DDPA over the same energy range. This indicates that the increase in total energy dissipation was not directly proportional to the expansion of the projected delamination area. Therefore, the projected delamination area alone may not fully represent the overall damage severity under relatively high-energy impacts.

To further examine the internal damage mechanisms, the specimen subjected to the 25 J impact was selected for μCT and cross-sectional optical microscopy observations, as shown in Figure 9b,c. The μCT results revealed that damage was concentrated around the impact center and gradually decreased with increasing distance from the impact location. The dominant damage modes were interlaminar delamination and intralaminar matrix cracking. Delamination occurred mainly at interfaces between UD plies and exhibited fan-shaped or approximately elliptical morphologies, whereas matrix cracks were primarily distributed within the UD plies.

The cross-sectional optical microscopy observations further showed that no extensively fragmented region developed beneath the impact center. Matrix damage was characterized mainly by shear and transverse cracks, while the opening of interlaminar delamination remained relatively limited. Except for the back-surface WF ply, no obvious fiber fracture was observed within the UD plies. Overall, the internal damage under the 25 J impact was dominated by matrix cracking and interlaminar delamination, consistent with the macroscopic observation that no through-thickness failure occurred.

## 4. Progressive Damage Model for UD/WF Laminates

To characterize the intralaminar damage and interlaminar delamination of UD/WF laminates under low-velocity impact, a progressive damage model was developed. Continuum damage mechanics (CDM) was employed to describe intralaminar damage in the UD and WF plies, while a cohesive zone model (CZM) was used to characterize delamination at the interfaces between adjacent plies.

### 4.1. Intralaminar Progressive Damage Model

#### 4.1.1. Damage Constitutive Framework

Within the framework of continuum damage mechanics (CDM), damage variables were introduced to describe the progressive degradation of material stiffness. For a three-dimensional stress state, the nominal stress in the damaged material is related to the effective stress in the equivalent undamaged material by

(1) $$\bar{\sigma }=M(d)\sigma$$

where *M*(*d*) is the damage-effect tensor defined in terms of the damage variables *d*.

Based on the strain-equivalence principle proposed by Lemaitre [29], the constitutive relation of the damaged material can be expressed as

(2) $$\sigma =C^{d}(d)\varepsilon$$

where *C^d^* is the damaged stiffness matrix, which is related to the initial undamaged stiffness matrix *C*^0^ through:

(3) $$C^{d}=M^{-1}:C^{0}$$

Considering the differences in material architecture and dominant failure modes between the UD and WF plies, separate intralaminar damage formulations were established. For the UD plies, four failure modes were considered: fiber tension (*d_ft_*), fiber compression (*d_fc_*), matrix tension (*d_mt_*), and matrix compression (*d_mc_*). For the WF plies, five failure modes were considered: warp tension (*d_wt_*), warp compression (*d_wc_*), weft tension (*d_ft_*), weft compression (*d_fc_*), and in-plane shear (*d_s_*). All damage variables range from 0 to 1, where 0 represents the undamaged state and 1 corresponds to complete failure of the associated damage mode.

#### 4.1.2. Damage Model for UD Plies

(1) Damage initiation criteria

Fiber tensile failure is assumed to initiate when

(4) $$\frac{\sigma_{11}}{X_{t}}\geq 1$$

whereas fiber compressive failure is governed by

(5) $$\frac{-\sigma_{11}}{X_{c}}\geq 1$$

**Figure 10:**
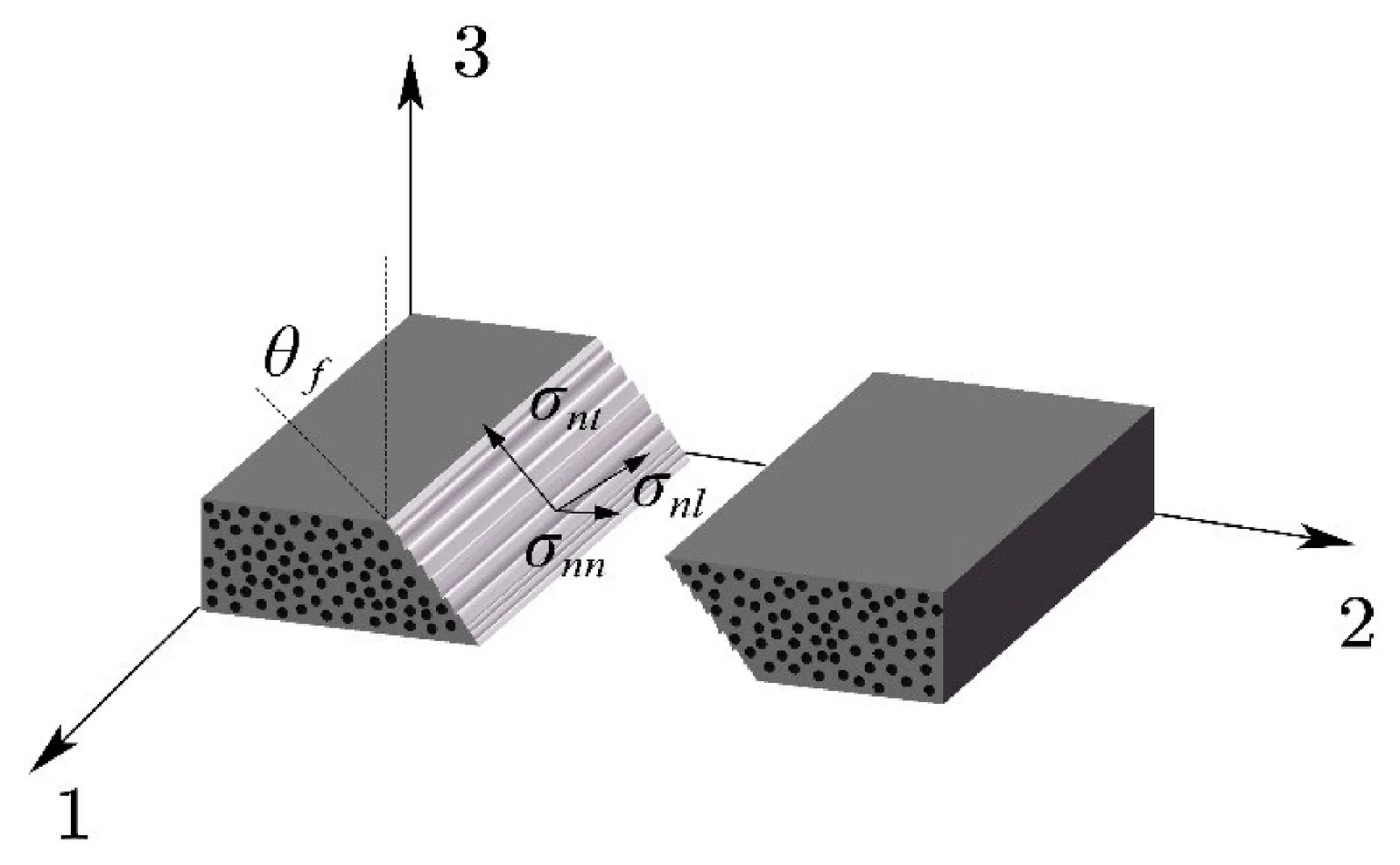
Stress components acting on the potential fracture plane, where 1, 2, and 3 denote the fiber, in-plane transverse, and through-thickness directions, respectively.

Matrix damage is evaluated using the LaRC05 failure criterion. By introducing a potential fracture plane, as illustrated in Figure 10, matrix damage initiation is determined from the combination of normal and shear stresses acting on the fracture plane:

(6) $${\left(\frac{\tau_{nt}}{S_{t}-\mu_{t}\sigma_{nn}}\right)}^{2}+{\left(\frac{\tau_{nl}}{S_{l}-\mu_{l}\sigma_{nn}}\right)}^{2}+{\left(\frac{\langle \sigma_{nn}\rangle }{Y_{t}}\right)}^{2}=1$$

Here, *σ*_11_ is the stress along the fiber direction, and *X_t_* and *X_c_* are the longitudinal tensile and compressive strengths. *σ_nn_*, *σ_nt_* and *σ_nl_* denote the normal and shear stress components acting on the potential fracture plane; *μ_t_* and *μ_l_* are the corresponding friction coefficients; *S_t_*, *S_l_* and *Y_t_* are the associated strength parameters. The Macaulay brackets are defined as ⟨x⟩ = max(0, x), allowing the criterion to account for the different effects of tensile and compressive normal stresses on matrix failure. The fracture-plane angle *θ_f_* was searched numerically from 0° to 180° in 1° increments, and the angle corresponding to the maximum LaRC05 failure index was identified as the critical fracture-plane angle.

(2) Damage evolution law

Once the fiber damage initiation criterion is satisfied, a fracture-energy-based damage evolution law is employed to describe the progressive stiffness degradation. The fiber damage variable is expressed as

(7) $$d_{i}=\frac{\varepsilon_{i}^{f}}{\varepsilon_{i}^{f}-\varepsilon_{i}^{o}}\left(1-\frac{\varepsilon_{i}^{o}}{\varepsilon_{i}}\right)(i=ft,fc)$$

where $\varepsilon_{i}$ (*i* = *ft*, *fc*) is the elastic strain associated with failure mode *i*, and $\varepsilon_{i}^{f}$ is the final failure strain corresponding to *d_i_* = 1. The final failure strains for fiber tension and compression are determined from the corresponding fracture toughness and characteristic element length as

(8) $$\varepsilon_{ft}^{f}=\frac{2G_{ft}}{X_{t}L_{c}}$$

(9) $$\varepsilon_{fc}^{f}=\frac{2G_{fc}}{X_{c}L_{c}}$$

where *G_i_* (*i* = *ft*, *fc*) is the fracture toughness of the corresponding failure mode, and *L_c_* is the characteristic element length.

For matrix damage, once the LaRC05 initiation criterion is satisfied, damage evolution is driven by the equivalent strain on the potential fracture plane [30]:

(10) $$d_{mk}=\frac{\varepsilon_{eq}^{f}}{\varepsilon_{eq}^{f}-\varepsilon_{eq}^{o}}\left(1-\frac{\varepsilon_{eq}^{o}}{\varepsilon_{eq}}\right)(k=t,c)$$

where the equivalent strain is defined as:

(11) $$\varepsilon_{eq}=\sqrt{{\langle \varepsilon_{nn}\rangle }^{2}+\varepsilon_{nt}^{2}+\varepsilon_{nl}^{2}}$$

Here, *ε_nn_*, *ε_nt_*, and *ε_nl_* are the strain components on the potential fracture plane. $\varepsilon_{eq}^{o}$ is determined at damage initiation according to the LaRC05 criterion, while $\varepsilon_{eq}^{f}$ is determined from the equivalent stress at damage initiation and the corresponding matrix fracture toughness. The subscripts *k* = *t*, *c* denote tensile and compressive matrix damage.

#### 4.1.3. Damage Model for WF Plies

Considering the interlaced warp–weft architecture of the WF plies, the damage formulation was established based on the models proposed by Mendes et al. [31], Iannucci et al. [32], and Xiong et al. [33]. Damage in the two principal fabric directions and in-plane shear damage were considered, with tensile and compressive failure treated separately for the warp and weft directions.

(1) Damage initiation criteria

Tensile and compressive damage in the warp and weft directions is determined using the corresponding stress-based failure criterion:

(12) $$\frac{\sigma_{ii}}{S_{ii}^{k}}\geq 1(k=t,c;i=1,2)$$

where *k* = *t*, *c* denote tensile and compressive failure; *i* = 1, 2 denote the two principal fabric directions; *σ_ii_* is the current normal stress in direction *i*; $S_{ii}^{k}\;$ is the strength in the *i*-direction for failure mode *k*.

The initiation of in-plane shear damage is governed by

(13) $$\frac{\left|\tau_{12}\right|}{S_{12}}\geq 1$$

where *τ*_12_ is the in-plane shear stress and *S*_12_ is the corresponding shear strength.

(2) Damage evolution law

After the corresponding damage initiation criterion is satisfied in either of the two principal fabric directions, stiffness degradation is described using the same form of fracture-energy-based damage evolution law as that adopted above. The final failure strains are determined from the corresponding material strengths, fracture toughnesses, and characteristic element length:

(14) $$\varepsilon_{wt}^{f}=\frac{2G_{wt}}{S_{11}^{t}L_{c}}$$

(15) $$\varepsilon_{wc}^{f}=\frac{2G_{wc}}{S_{11}^{c}L_{c}}$$

(16) $$\varepsilon_{ft}^{f}=\frac{2G_{ft}}{S_{22}^{t}L_{c}}$$

(17) $$\varepsilon_{fc}^{f}=\frac{2G_{fc}}{S_{22}^{c}L_{c}}$$

Here, $S_{ii}^{k}$ is the strength in material direction *i* associated with failure mode *k* (*k* = *t*, *c*; *i* = 1, 2); *G_i_* denotes the fracture toughness associated with the corresponding failure mode; *L_c_* is the characteristic element length.

Once the in-plane shear damage initiation criterion is satisfied, the damage evolution is described using an elastic-shear-strain-based formulation [32]:

(18) $$d_{s}=\frac{\gamma_{12}^{f}}{\gamma_{12}^{f}-\gamma_{12}^{o}}\left(1-\frac{\gamma_{12}^{o}}{\gamma_{12}^{el}}\right)$$

(19) $$\gamma_{12}^{f}=\frac{2G_{s}}{S_{12}L_{c}}$$

where *d_s_* is the in-plane shear damage variable; $\gamma_{12}^{o}$ is the elastic shear strain at damage initiation; $\gamma_{12}^{el}$ is the current elastic shear strain; and $\gamma_{12}^{f}$ is the final elastic shear strain corresponding to complete shear failure (*d_s_* = 1), *G_s_* denotes the shear fracture toughness.

### 4.2. Interlaminar Damage Model

A cohesive zone model (CZM) was employed to describe delamination at the interfaces between adjacent plies. Because the resin-rich interlaminar region is relatively thin, a surface-based cohesive formulation was adopted to represent the interfacial behavior. The delamination process was described through a traction–separation law, a damage initiation criterion, and a damage evolution law.

#### 4.2.1. Traction–Separation Constitutive Relation

Before the onset of interfacial damage, the interface was assumed to follow an uncoupled linear elastic traction–separation relation:

(20) $$t=\left\{\begin{array}{c} t_{n}\\ t_{s}\\ t_{t} \end{array}\right\}=\left[\begin{array}{ccc} K_{n} & 0 & 0\\ 0 & K_{s} & 0\\ 0 & 0 & K_{t} \end{array}\right]\left\{\begin{array}{c} \delta_{n}\\ \delta_{s}\\ \delta_{t} \end{array}\right\}=K\delta$$

where *t_n_*, *t_s_*, *t_t_* and are the normal and two shear traction components, respectively. *δ_n_*, *δ_s_*, and *δ_t_* are the corresponding separation components. Equal penalty stiffnesses were assigned in the three directions [34], i.e., *K_n_* = *K_s_* = *K_t_*.

#### 4.2.2. Damage Initiation Criterion

Interfacial damage initiation was determined using the quadratic stress criterion [34]:

(21) $${\left(\frac{\langle t_{n}\rangle }{N}\right)}^{2}+{\left(\frac{t_{s}}{S}\right)}^{2}+{\left(\frac{t_{t}}{T}\right)}^{2}=1$$

where *N* is the interfacial normal tensile strength, *S* and *T* are the shear strengths in the two tangential directions, respectively. Since compressive normal traction does not initiate delamination, the Macaulay brackets are applied to the normal traction term.

#### 4.2.3. Damage Evolution Law

After the initiation of interfacial damage, delamination propagation was described by linear stiffness degradation:

(22) $$K^{d}=\left(1-d\right)K$$

where *K* and *K^d^* denote the initial and damaged interfacial stiffness matrices, respectively, and *d* is the interfacial damage variable.

**Figure 11:**
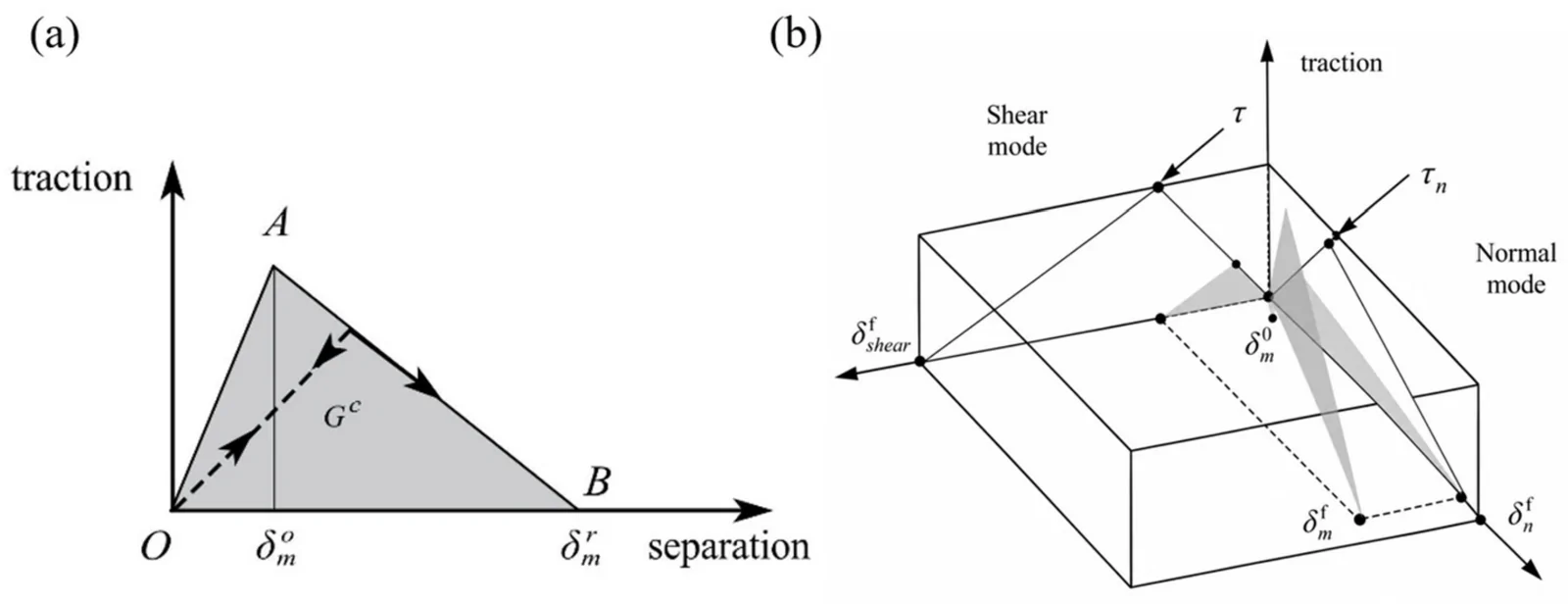
Schematic of interlaminar damage evolution [35]: (**a**) linear damage evolution; and (**b**) mixed-mode traction–separation response.

An energy-based linear damage evolution law was adopted to describe interfacial degradation under mixed-mode loading. The corresponding traction–separation responses are illustrated in Figure 11.

The damage variable is determined from the equivalent separation (*δ_m_*) as

(23) $$d=\frac{\delta_{m}^{f}\left(\delta_{m}-\delta_{m}^{o}\right)}{\delta_{m}\left(\delta_{m}^{f}-\delta_{m}^{o}\right)}$$

where the equivalent separation(*δ_m_*) is defined as

(24) $$\delta_{m}=\sqrt{\delta_{n}+{\delta_{s}}^{2}+{\delta_{t}}^{2}}$$

Here, $\delta_{m}^{o}$ and $\delta_{m}^{f}$ denote the equivalent separations at damage initiation and complete interfacial failure.

For mixed-mode delamination, the critical energy release rate was determined using the Benzeggagh–Kenane (B–K) criterion:

(25) $$G_{C}=G_{IC}+\left(G_{IIC}-G_{IC}\right){\left(\frac{G_{II}}{G_{T}}\right)}^{\eta }$$

where *G_c_* is the critical energy release rate under mixed-mode loading; *G_Ic_* and *G_IIc_* are the Mode I and Mode II interlaminar fracture toughnesses; *G_I_*, *G_II_*, and *G_III_* are the Mode I, Mode II, and Mode III energy release rates, respectively; *η* is the material parameter in the B–K criterion.

## 5. Finite Element Simulation and Discussion of Low-Velocity Impact Behavior

### 5.1. Finite Element Model

**Figure 12:**
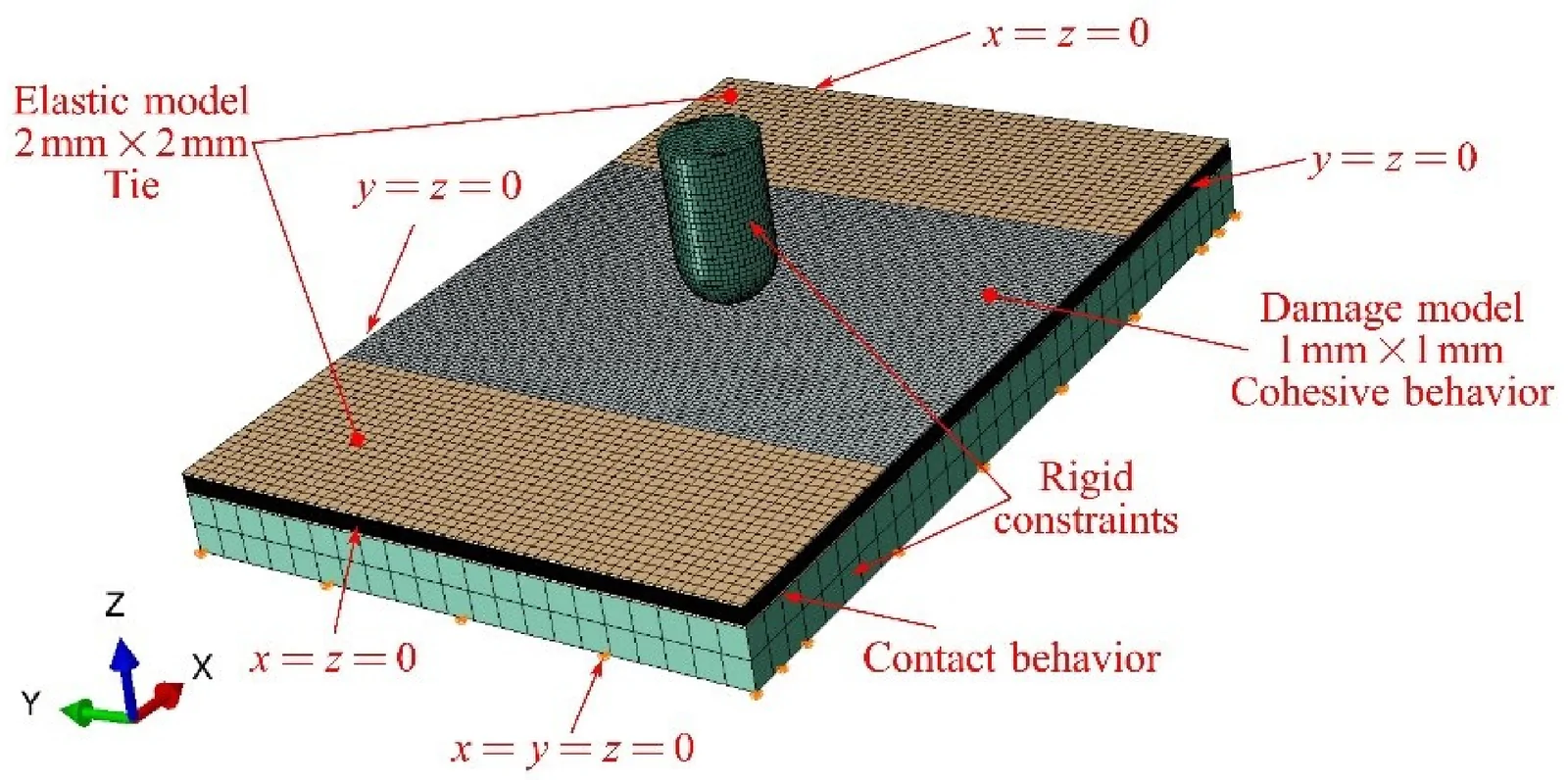
Finite element model of the UD/WF laminate subjected to low-velocity impact.

Based on the progressive damage model developed in Section 4, the intralaminar damage of the UD and WF plies was implemented in Abaqus/Explicit 2024 through a user-defined VUMAT subroutine, while interlaminar delamination was modeled using cohesive contact. The finite element model, shown in Figure 12, consisted of the impactor, fixture, and UD/WF laminate. The impactor and fixture were modeled as rigid bodies, whereas the laminate was discretized using SC8R continuum shell elements [36]. A locally refined finite element discretization was adopted for the impact region following commonly used modeling strategies for low-velocity impact simulations of composite laminates [37]. A refined mesh with an element size of 1 mm × 1 mm was applied to a 75 mm × 100 mm region centered on the impact location, while a mesh size of 2 mm × 2 mm was used in the remaining region. The actual ply thicknesses were assigned in the model, with thicknesses of 0.142 and 0.422 mm for the UD and WF plies, respectively.

The 1 mm × 1 mm mesh size in the refined impact region was selected with reference to published mesh-sensitivity studies on low-velocity impact simulations of composite laminates [38,39,40]. Zhou et al. [38] investigated element sizes of 0.8, 1.0, and 1.2 mm and adopted a 1 mm × 1 mm mesh to balance progressive-damage prediction accuracy and computational efficiency. Mohsin et al. [39] reported maximum loads of 9.6, 9.7, and 8.7 kN for element sizes of 0.5, 1.0, and 2.0 mm, respectively, with corresponding computational times of 34.5, 3.0, and 0.6 h. Thus, the 1 mm mesh provided a peak-load prediction comparable to the finer 0.5 mm mesh while substantially reducing the computational cost. Panettieri et al. [40] further investigated cohesive-element mesh sizes of 0.5 and 1.0 mm and highlighted the influence of mesh resolution on delamination prediction and computational performance. Based on these studies, a 1 mm × 1 mm mesh was adopted in the refined impact region as a practical compromise between damage resolution and computational efficiency. In addition, the fracture-energy-based damage evolution laws incorporate the characteristic element length, which regularizes the strain-softening response and reduces mesh sensitivity. The adopted discretization was further supported by the good agreement between the numerical and experimental results in terms of peak load, projected delamination area, and indentation depth.

Regarding computational cost, the simulations were performed on a workstation equipped with an Intel Core i9-12900K processor (3.20 GHz, 16 cores). With the adopted local mesh-refinement strategy, a typical impact simulation required approximately 15 h of wall-clock time.

Cohesive contact was defined at the interfaces between adjacent plies within the refined impact region, whereas a tie constraint was applied between adjacent UD plies with the same fiber orientation [36]. The coefficient of friction between the specimen and fixture was set to 0.2 [23]. The impactor and fixture were modeled as rigid bodies, with reference points (RPs) defined for the corresponding rigid-body motions. The fixture RP was fully constrained in all translational and rotational degrees of freedom. To reproduce the restraint provided by the experimental fixture and clamps, fixed translational displacement constraints (U_1_ = U_2_ = U_3_ = 0) were applied to the specimen boundary regions corresponding to the clamped/support regions. The impactor mass of 5.607 kg was assigned to the impactor RP. Impact loading was introduced by prescribing an initial velocity normal to the laminate surface rather than by applying an external force. According to E = 1/2 mv^2^, initial velocities of 1888.6, 2462.5, and 2986.2 mm/s were applied for the 10, 17, and 25 J impact cases, respectively. The other translational degrees of freedom of the impactor were constrained so that its motion was restricted to the impact direction.

The intralaminar fracture toughnesses of the UD and WF plies, together with the parameters for the cohesive damage model and LaRC05 failure criterion, were adopted from Refs. [19,31,32,34,37] and are summarized in Table 4, Table 5 and Table 6.

### 5.2. Numerical Results and Discussion

To evaluate the predictive capability of the finite element model, the calculated impact responses, indentation depths, and delamination damage were first compared with the experimental results. The validated model was then used to further investigate the distributions of interlaminar and intralaminar damage under the 25 J impact condition.

#### 5.2.1. Model Validation

(1) Impact response

**Figure 13:**
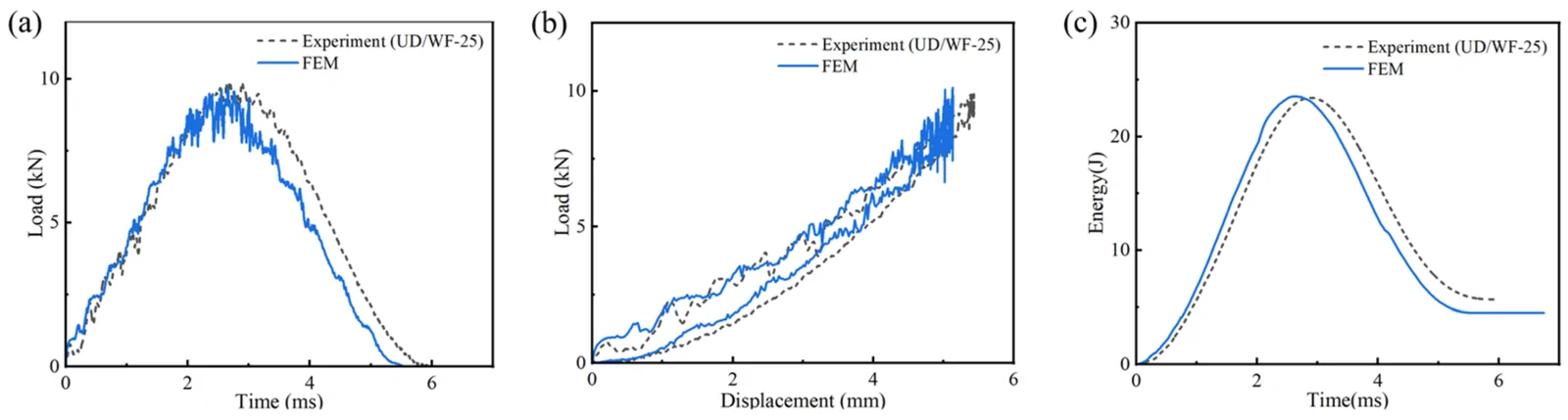
Comparison between experimental and numerical impact responses: (**a**) load–time curves; (**b**) load–displacement curves; and (**c**) energy–time curves.

Taking the 25 J impact case as an example, Figure 13 compares the calculated load–time, load–displacement, and energy–time responses with the corresponding experimental results. The numerical model reproduces the overall trends of the experimental curves reasonably well.

A quantitative comparison of the characteristic response parameters at different impact energies is provided in Table 7. The prediction errors for the peak load, contact duration, and maximum displacement were all below 8%, with the error in peak load at 25 J being only 0.40%. The dissipated energy was underestimated, with errors ranging from approximately 12% to 15% and reaching 14.82% at 25 J. This systematic underprediction may be attributed to physical dissipation mechanisms and modeling idealizations that are not fully represented in the numerical model. Although the principal intralaminar and interlaminar damage mechanisms were captured, material damping and some microscale damage processes were not explicitly represented, which may result in lower predicted energy dissipation. In addition, tie constraints were applied between adjacent UD plies with the same fiber orientation, thereby excluding possible interfacial damage and the associated energy dissipation at these interfaces, which may further contribute to the underprediction. Overall, the model provides satisfactory predictions of the global impact response of the UD/WF laminates.

Overall, the model provides satisfactory predictions of the global impact response of the UD/WF laminates.

(2) Indentation and delamination damage

**Figure 14:**
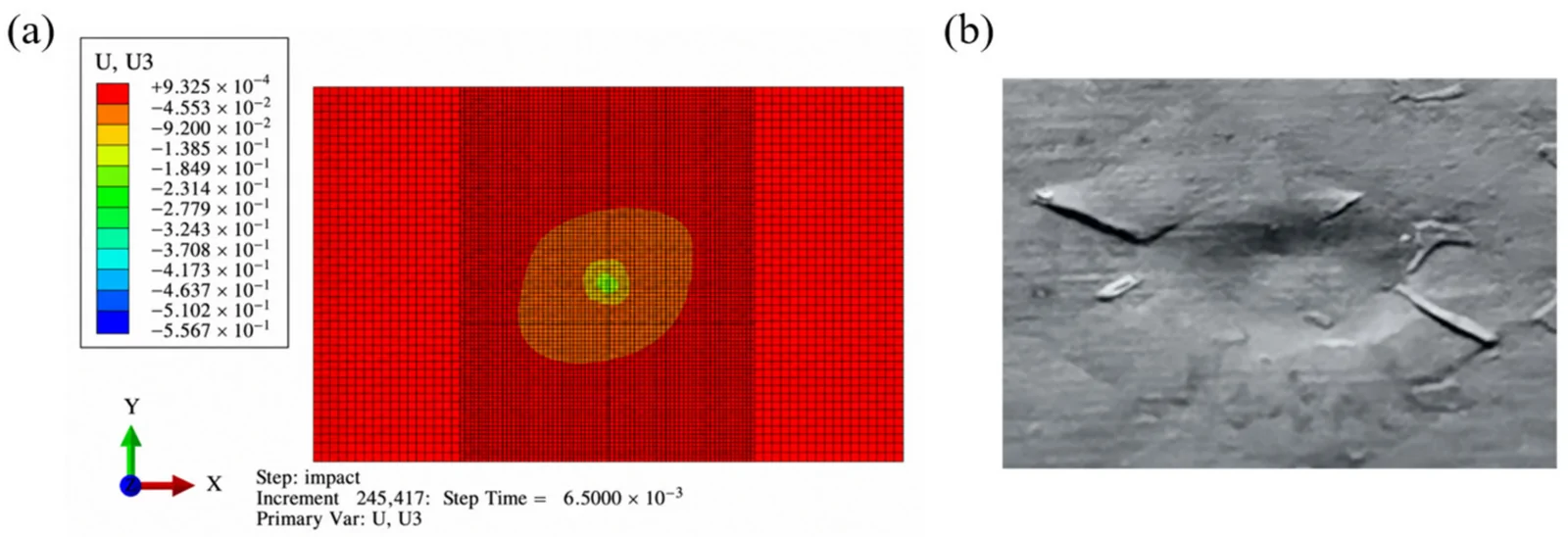
Comparison between experimental and numerical indentation morphology under the 25 J impact condition. (**a**) out-of-plane displacement contour; and (**b**) experimental indentation.

Figure 14 compares the numerically predicted out-of-plane displacement field after impact with the experimentally observed indentation morphology. Both exhibit an approximately circular indentation centered on the impact location. The calculated indentation depths and projected delamination damage areas (DDPAs) at different impact energies are compared with the experimental measurements in Table 8. The numerical DDPA was determined using the interfacial damage variable (d) defined in Equation (23). Regions with (d = 1.0), corresponding to complete interfacial failure, were classified as fully delaminated. The fully delaminated region at each cohesive interface was extracted separately and orthogonally projected along the through-thickness direction onto the laminate plane. The projected regions from all cohesive interfaces were then combined by taking their geometric union, and the area of the resulting union was defined as the numerical DDPA. Thus, overlapping delaminated regions at different interfaces were counted only once.

The maximum error in DDPA was 6.72%, while the error in indentation depth did not exceed 10%. These results demonstrate that the model can predict not only the global impact response but also the permanent indentation and the extent of delamination damage with reasonable accuracy.

#### 5.2.2. Damage Distribution Characteristics

(1) Interlaminar delamination

**Figure 15:**
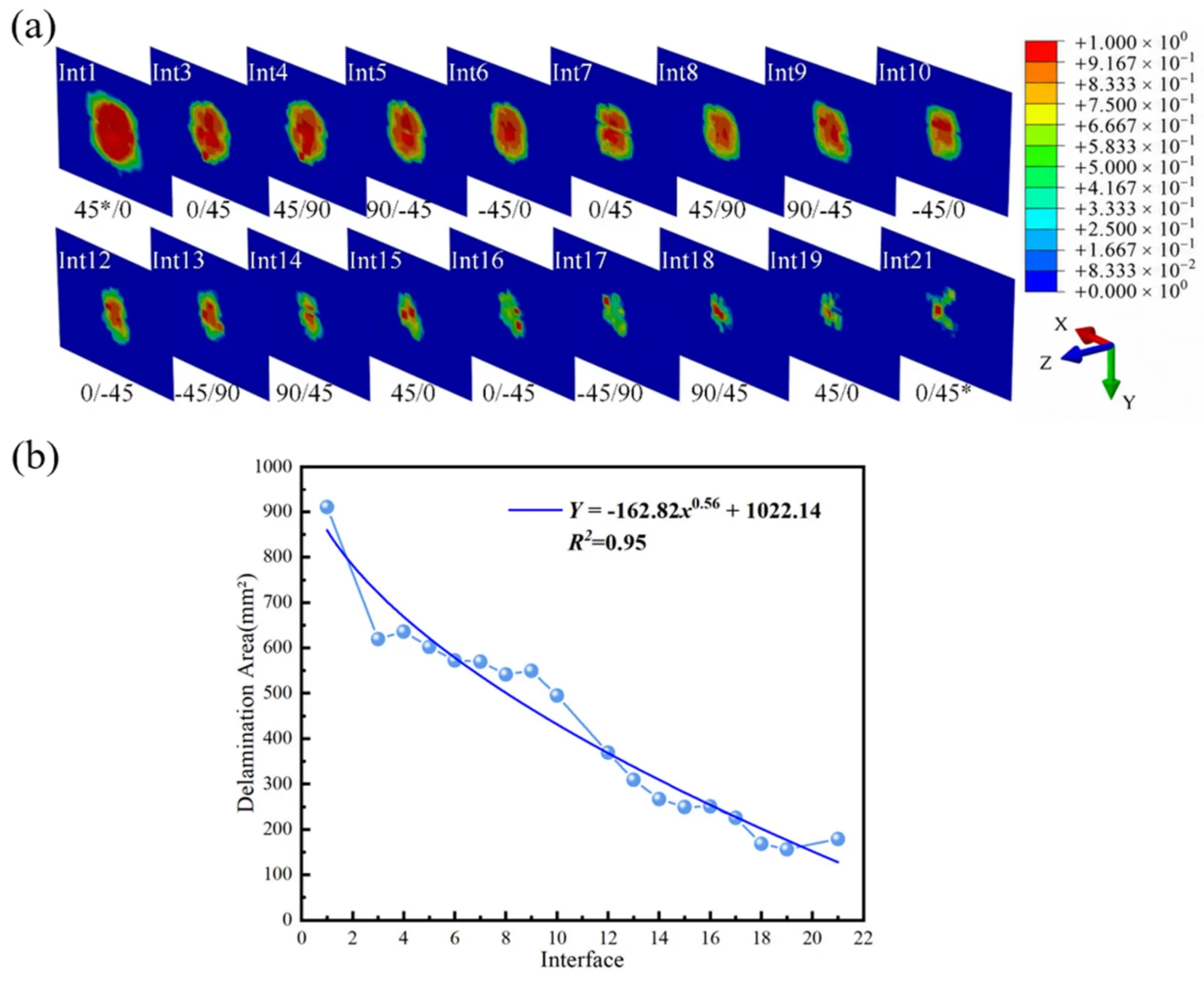
Interlaminar delamination in the UD/WF laminate under a 25 J impact: (**a**) delamination distributions at different interfaces; and (**b**) variation in delamination area through the laminate thickness.

In the finite element model, cohesive contact was assigned only to the interfaces where interlaminar delamination was allowed to develop. Int i denotes the physical interface between plies i and i + 1 (i = 1, …, 21). Int 2, Int 11, and Int 20 correspond to the 0/0 ply pairs between plies 2–3, 11–12, and 20–21, respectively, and were modeled using tie constraints; therefore, these three interfaces were excluded from the delamination-area analysis. The remaining 18 interfaces were modeled using cohesive contact. Figure 15 shows the delamination distributions at different ply interfaces under the 25 J impact. Delamination was primarily concentrated near the impact center and exhibited an approximately circular distribution. Its extent generally decreased through the laminate thickness, with a pronounced reduction observed around Int 10–Int 12.

As shown in Figure 15b, the delamination area generally exhibited a power-law decay trend through the laminate thickness and was fitted by Y = −162.82X^0.56^ + 1022.14, R^2^ = 0.95, where Y represents the delamination area at a given interface in mm^2^ and X denotes the physical interface index from the impact side (Int 2, Int 11, and Int 20 were excluded because they were modeled using tie constraints). The maximum delamination area occurred at Int 1 and reached 910.29 mm^2^. These results indicate that, under the 25 J impact, delamination is concentrated primarily at interfaces near the impact surface and exhibits a pronounced nonuniform attenuation through the laminate thickness.

(2) Damage in the UD plies

**Figure 16:**
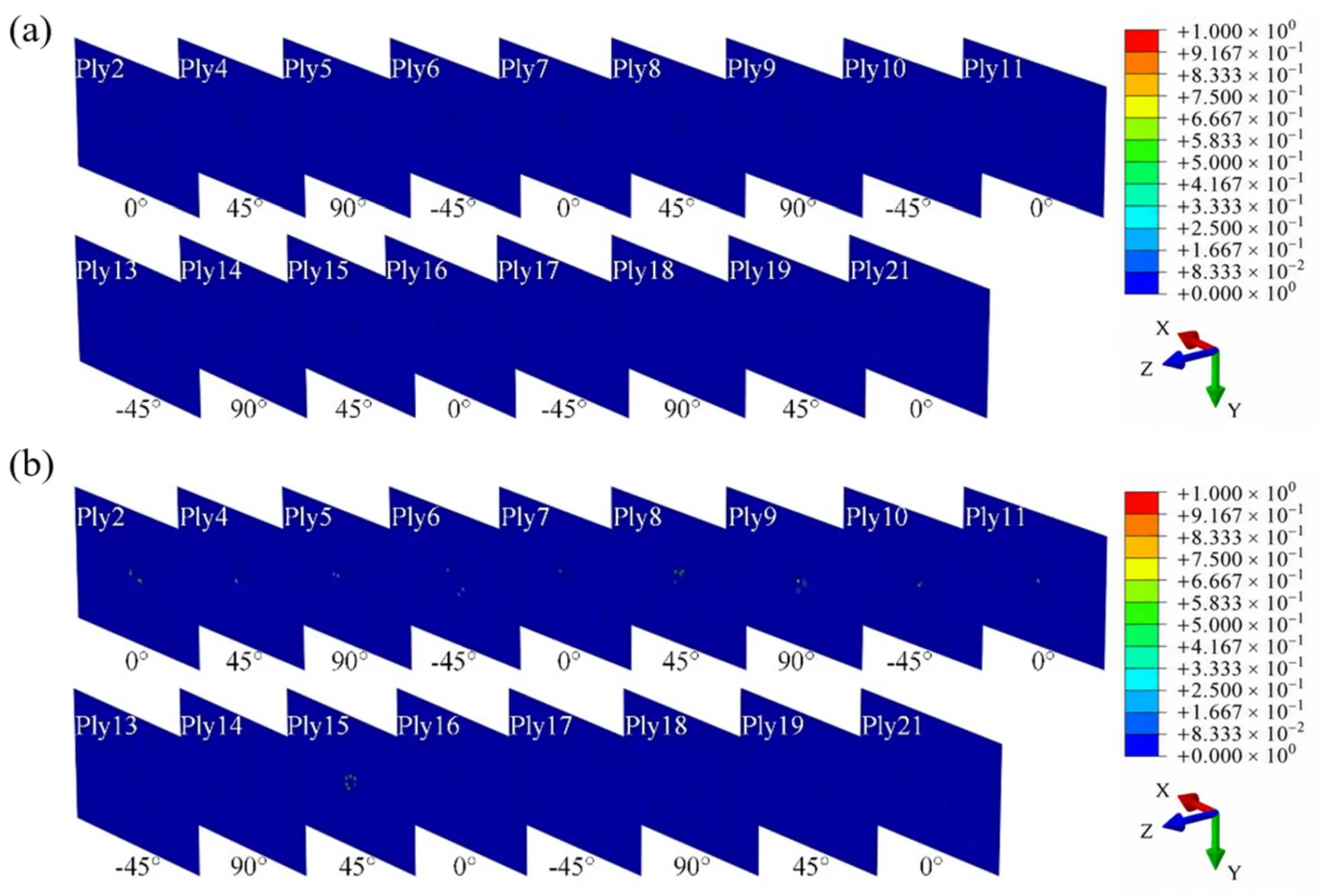
Fiber damage distributions in the UD plies under a 25 J impact: (**a**) fiber tensile damage; and (**b**) fiber compressive damage.

**Figure 17:**
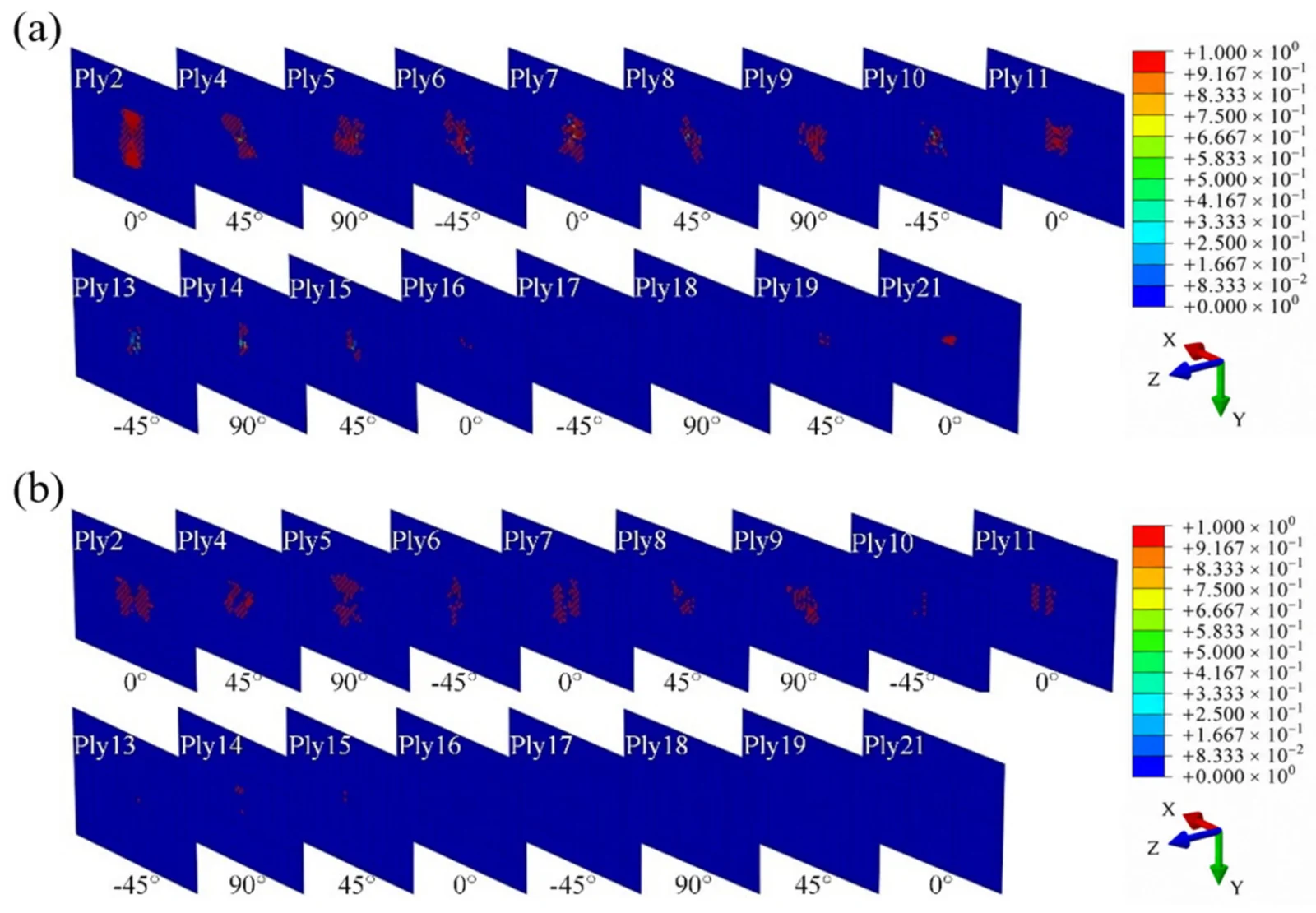
Matrix damage distributions in the UD plies under a 25 J impact: (**a**) matrix tensile damage; and (**b**) matrix compressive damage.

Figure 16 and Figure 17 show the predicted fiber and matrix damage, respectively, in the UD plies under the 25 J impact. Both fiber tensile and compressive damage remained relatively limited, indicating that the internal UD plies largely retained their fiber load-bearing integrity. This finding is consistent with the cross-sectional microscopic observations, in which no obvious extensive fiber fracture was detected in the UD plies.

In contrast, matrix damage was considerably more pronounced. Matrix tensile damage exhibited predominantly band-like distributions along the fiber directions, whereas matrix compressive damage was concentrated around the impact location. These results indicate that the intralaminar damage in the UD plies under the 25 J impact was dominated by matrix damage, while fiber damage remained comparatively limited.

(3) Damage in the WF plies

**Figure 18:**
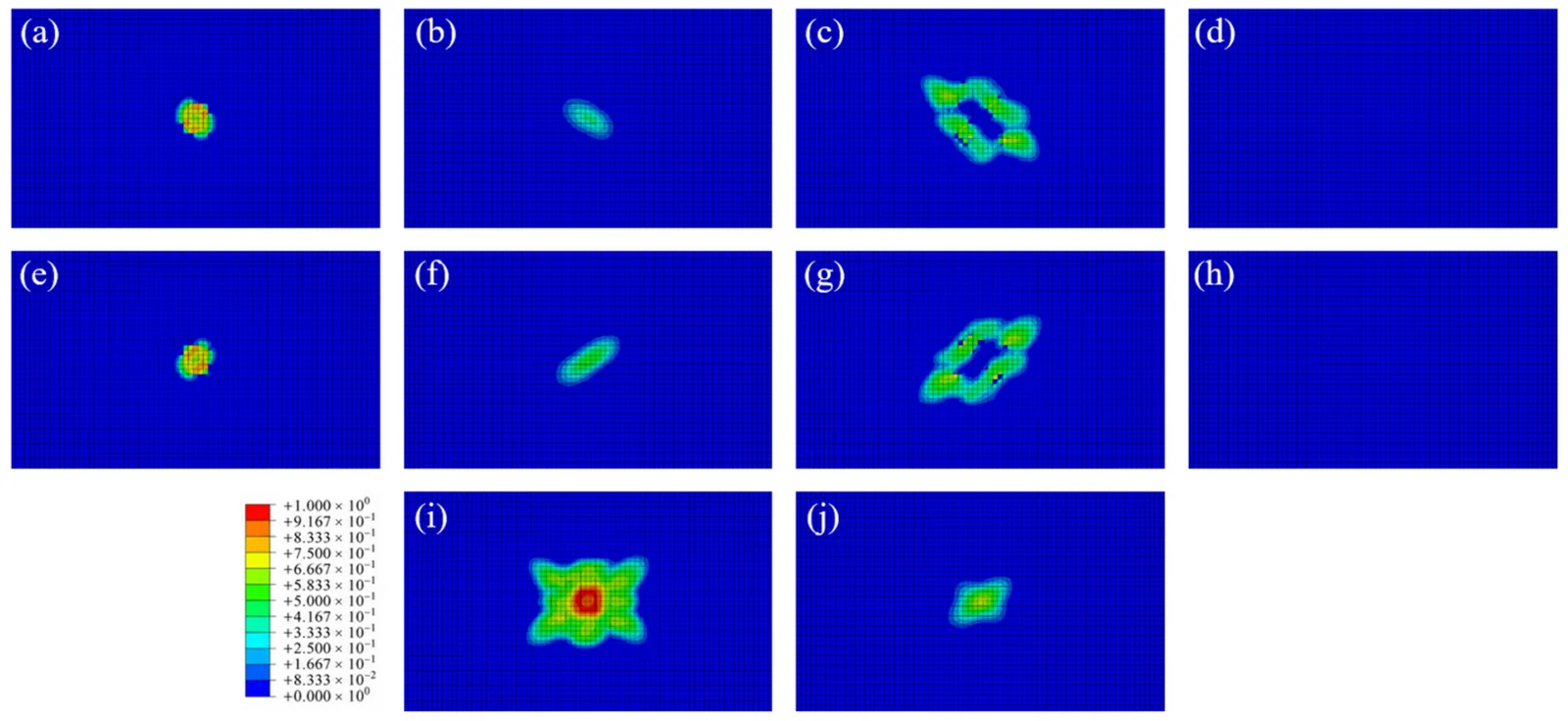
Damage distributions in the WF plies under a 25 J impact: (**a**–**h**) tensile and compressive damage in the warp and weft directions; and (**i**,**j**) in-plane shear damage.

Figure 18 shows the damage distributions in the impact-side Ply 1 and back-side Ply 22 of the woven-fabric layers. Pronounced compressive damage in both the warp and weft directions was observed in the impact-side WF ply, whereas the back-side WF ply was dominated by tensile damage in the warp and weft directions. This difference reflects the distinct stress states experienced by the two surface plies during impact-induced bending.

In-plane shear damage was also pronounced in both WF surface plies and was concentrated around the impact region. The warp- and weft-direction damage exhibited intersecting patterns aligned with the fabric directions, which is generally consistent with the warp- and weft-oriented radial surface cracks observed experimentally. These results indicate that the damage response of the surface WF plies is governed jointly by their interlaced fabric architecture and the bending-induced stress state during impact.

#### 5.2.3. Correlation Between Experimental and Numerical Results

The combined experimental and numerical results demonstrate that the damage evolution of the UD/WF laminates cannot be adequately characterized by a single damage metric. When the impact energy increased from 17 to 25 J, the experimentally measured dissipated energy increased from 2.67 to 5.33 J, whereas the projected delamination area increased only from 923.78 to 987.70 mm^2^. This disparity indicates that the increase in total energy dissipation at higher impact energies is not accompanied by a proportional increase in the in-plane extent of delamination.

Combined with the μCT, cross-sectional microscopic observations, and finite element results at 25 J, the damage at this energy level involves not only an increase in delamination extent but also the development of intralaminar matrix damage and nonuniform delamination among different interfaces. Therefore, at relatively high impact energies, the increased damage severity is more likely associated with the concurrent development of multiple damage modes rather than a continued substantial expansion of the projected delamination area.

The numerical results further reveal distinct damage responses in the UD and WF plies. Fiber damage in the UD plies remained limited, whereas matrix tensile and compressive damage was more pronounced, consistent with the microscopic observations showing predominantly matrix cracking and interlaminar delamination without extensive fiber fracture in the UD plies. In contrast, the surface WF plies exhibited pronounced tensile/compressive damage in the warp and weft directions together with in-plane shear damage, whose spatial distributions were generally consistent with the warp- and weft-oriented radial cracks observed experimentally. These findings demonstrate that the UD and WF plies exhibit distinct impact damage responses and that the interaction between intralaminar damage and interlaminar delamination constitutes the multiscale impact damage pattern of the hybrid laminate.

## 6. Conclusions

Low-velocity impact tests at 10, 17, and 25 J, together with finite element simulations and multiscale damage characterization, were conducted to investigate the impact response and damage behavior of hybrid unidirectional-tape/woven-fabric (UD/WF) laminates. The main conclusions are summarized as follows:

(1) The low-velocity impact response of the UD/WF laminates exhibited a pronounced dependence on impact energy. The Hertzian failure load varied only slightly with increasing impact energy, whereas the peak load, maximum displacement, and dissipated energy increased markedly, reaching 10.08 kN, 5.51 mm, and 5.33 J, respectively, at 25 J. No pronounced abrupt load drop was observed at any of the investigated energy levels, indicating that the laminates retained their overall structural integrity within the tested impact-energy range.
(2) Impact damage became progressively more severe with increasing impact energy, although different damage metrics exhibited distinct energy-dependent trends. Surface damage evolved from localized indentation to radial cracking along the warp and weft directions of the woven fabric. The projected delamination damage area increased from 484.15 mm^2^ at 10 J to 923.78 mm^2^ at 17 J, but increased only slightly further to 987.70 mm^2^ at 25 J, indicating that the in-plane propagation of delamination tended to slow at higher impact energies. μCT and cross-sectional microscopic observations further showed that the internal damage at 25 J was concentrated near the impact region and was dominated by interlaminar delamination and matrix cracking, with no extensive fiber fracture observed in the UD plies.
(3) The developed progressive damage finite element model provided satisfactory predictions of both the global impact response and damage extent of the UD/WF laminates. The errors in peak load, contact duration, and maximum displacement were below 8%, while those in projected delamination area and indentation depth did not exceed 10%. The dissipated energy, however, was underestimated by approximately 12–15%. The numerical results further revealed a pronounced nonuniform attenuation of delamination through the laminate thickness under the 25 J impact. Matrix damage dominated the intralaminar damage in the UD plies, whereas fiber damage remained limited. In the surface WF plies, the predicted warp- and weft-direction damage patterns were consistent with the radial surface cracking observed experimentally.

The present progressive damage model incorporates the principal intralaminar and interlaminar damage mechanisms and is therefore expected to retain the capability to capture progressive damage evolution and the transition toward ultimate failure at impact energies beyond the experimentally investigated range. However, its quantitative predictive capability under such higher-energy conditions has not yet been experimentally validated. Future work will extend the impact-energy range beyond 25 J to determine the complete-failure threshold of the UD/WF laminates and further characterize the transition from progressive damage to severe fiber failure and penetration. In addition, higher-energy impact experiments will be conducted to further evaluate and validate the predictive capability of the present numerical model for damage evolution and ultimate laminate failure under higher-energy impact conditions.

## Figures and Tables

**Table 1:** Mechanical properties of the UD plies.

Property	Value
*E*_1_, *E*_2_, *E*_3_ (MPa)	124,000, 9500, 9500
*G*_12_, *G*_13_, *G*_23_ (MPa)	4200, 4200, 3300
*S*_12_, *S*_13_, *S*_23_ (MPa)	154, 154, 50
*ν*_12_, *ν*_13_, *ν*_23_	0.3, 0.3, 0.35
*ρ* (kg/m^3^)	1676
*X_t_*, *Y_t_*, *Z_t_* (MPa)	1900, 58, 58
*X_c_*, *Y_c_* (MPa)	829, 205

**Table 2:** Mechanical properties of the WF plies.

Property	Value
*E*_1_, *E*_2_, *E*_3_ (MPa)	56,000, 56,000, 10,000
*G*_12_, *G*_13_, *G*_23_ (MPa)	4200, 4200, 3500
*S*_12_, *S*_13_, *S*_23_ (MPa)	77, 80, 80
*ν*_12_, *ν*_13_, *ν*_23_	0.04, 0.35, 0.35
*ρ* (kg/m^3^)	1592
*X_t_*, *Y_t_*, *Z_t_* (MPa)	967, 967, 75
*X_c_*, *Y_c_* (MPa)	510, 510

**Table 3:** Characteristic response parameters of the UD/WF laminates at different impact energies.

Energy	Specimen	*F_H_* (kN)	*F_F_* (kN)	*E_D_* (J)	*T_D_* (ms)	*D_E_* (mm)
10 J	UD/WF	3.31 ± 0.17	5.47 ± 0.03	1.91 ± 0.18	6.38 ± 0.07	3.77 ± 0.02
17 J	UD/WF	3.31 ± 0.12	7.62 ± 0.14	2.67 ± 0.12	6.27 ± 0.11	4.81 ± 0.09
25 J	UD/WF	3.10 ± 0.09	10.08 ± 0.14	5.33 ± 0.90	5.86 ± 0.05	5.51 ± 0.06

**Table 4:** Intralaminar fracture toughness parameters for the UD plies [19,37].

Parameter	*G_ft_* (kJ/m^2^)	*G_fc_* (kJ/m^2^)	*G_mt_* (kJ/m^2^)	*G_mc_* (kJ/m^2^)
value	133	40	0.52	1.61

**Table 5:** Intralaminar fracture toughness parameters for the WF plies [31,32].

Parameter	*G_wt_* (kJ/m^2^)	*G_wc_* (kJ/m^2^)	*G_ft_* (kJ/m^2^)	*G_fc_* (kJ/m^2^)	*G_s_* (kJ/m^2^)
value	125	40	125	40	0.92

**Table 6:** Parameters for the cohesive damage model and LaRC05 failure criterion [19,34,37].

Parameter	*G_Ic_* (kJ/m^2^)	*G_IIc_* (kJ/m^2^)	*G_IIIc_* (kJ/m^2^)	*η*	*N* (MPa)	*S* (MPa)	*T* (MPa)	*K_n_*, *K_s_*, *K_t_* (N/mm^3^)	*μ_t_*	*μ_l_*
value	0.56	0.92	0.92	1.46	30	80	80	10^6^, 10^6^, 10^6^	0.29	0.76

**Table 7:** Comparison of experimental and numerical characteristic response parameters at different impact energies.

Energy	Value	*F_F_* (kN)	*E_D_* (J)	*T_D_* (ms)	*D_E_* (mm)
10 J	Experimental	5.47	1.91	6.38	3.77
	Calculated	5.18	1.68	6.36	3.61
	Error	5.30%	12.04%	0.31%	4.24%
17 J	Experimental	7.62	2.67	6.27	4.81
	Calculated	8.21	2.34	6.04	4.57
	Error	7.74%	12.36%	3.67%	4.99%
25 J	Experimental	10.08	5.33	5.86	5.51
	Calculated	10.12	4.54	5.82	5.13
	Error	0.40%	14.82%	0.68%	6.90%

**Table 8:** Comparison of experimental and numerical projected delamination areas and indentation depths at different impact energies.

Impact Energy	DDPA (mm^2^)			Indentation Depth (mm)		
	Calculated	Experimental	Error	Calculated	Experimental	Error
10 J	462.26	484.15	4.52%	0.09	0.10	10.00%
17 J	861.54	923.78	6.72%	0.12	0.13	7.69%
25 J	942.36	987.70	4.59%	0.15	0.16	6.25%

## Data Availability

The original contributions presented in this study are included in the article/Supplementary Materials. Further inquiries can be directed to the author.

## Supplementary Materials

The following supporting information can be downloaded at: https://www.mdpi.com/article/10.3390/polym18192414/s1, File S1: Supplementary Data.

## Abbreviations

The following abbreviations are used in this manuscript:

UD/WF	Unidirectional-tape/woven-fabric
UD	Unidirectional
WF	Woven fabric
FEM	Finite element method
CFRP	Carbon fiber-reinforced polymer
CDM	Continuum damage mechanics
CZM	Cohesive zone model
CT	Computed tomography
DDPA	Projected delamination damage area
